# C(*sp* ^n^)−X (n=1–3) Bond Activation by Palladium

**DOI:** 10.1002/chem.202103953

**Published:** 2022-01-31

**Authors:** Thomas Hansen, Xiaobo Sun, Marco Dalla Tiezza, Willem‐Jan van Zeist, Jordi Poater, Trevor A. Hamlin, F. M. Bickelhaupt

**Affiliations:** ^1^ Department of Theoretical Chemistry and Amsterdam Center for Multiscale Modeling (ACMM) Vrije Universiteit Amsterdam De Boelelaan 1083 1081 HV Amsterdam (The Netherlands; ^2^ Departament de Química Inorgànica i Orgànica & IQTCUB Universitat de Barcelona 08028 Barcelona Spain; ^3^ Leiden Institute of Chemistry Leiden University Einsteinweg 55 2333 CC Leiden (The Netherlands; ^4^ ICREA, Pg. Lluís Companys 23 08010 Barcelona Spain; ^5^ Institute for Molecules and Materials (IMM) Radboud University Heyendaalseweg 135 6525 AJ Nijmegen (The Netherlands

**Keywords:** activation strain model, density functional calculations, homogeneous catalysis, oxidative addition, reactivity

## Abstract

We have studied the palladium‐mediated activation of C(*sp* ^n^)−X bonds (n = 1–3 and X = H, CH_3_, Cl) in archetypal model substrates H_3_C−CH_2_−X, H_2_C=CH−X and HC≡C−X by catalysts PdL_n_ with L_n_ = no ligand, Cl^−^, and (PH_3_)_2_, using relativistic density functional theory at ZORA‐BLYP/TZ2P. The oxidative addition barrier decreases along this series, even though the strength of the bonds increases going from C(*sp* ^3^)−X, to C(*sp* ^2^)−X, to C(*sp*)−X. Activation strain and matching energy decomposition analyses reveal that the decreased oxidative addition barrier going from *sp* ^3^, to *sp* ^2^, to *sp*, originates from a reduction in the destabilizing steric (Pauli) repulsion between catalyst and substrate. This is the direct consequence of the decreasing coordination number of the carbon atom in C(*sp* ^n^)−X, which goes from four, to three, to two along this series. The associated net stabilization of the catalyst–substrate interaction dominates the trend in strain energy which indeed becomes more destabilizing along this same series as the bond becomes stronger from C(*sp* ^3^)−X to C(*sp*)−X.

## Graphical abstract

**Less is more**! Our quantum chemical analyses reveal that the activity of palladium catalysts towards C−X bond activation increases along ethane, ethylene, acetylene because the steric repulsion between catalyst and substrate is relieved as the number of substituents around the carbon atom goes down.

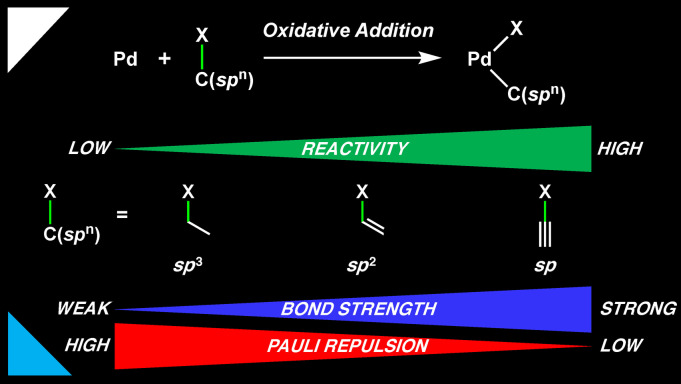

## Introduction

**Scheme 1:**
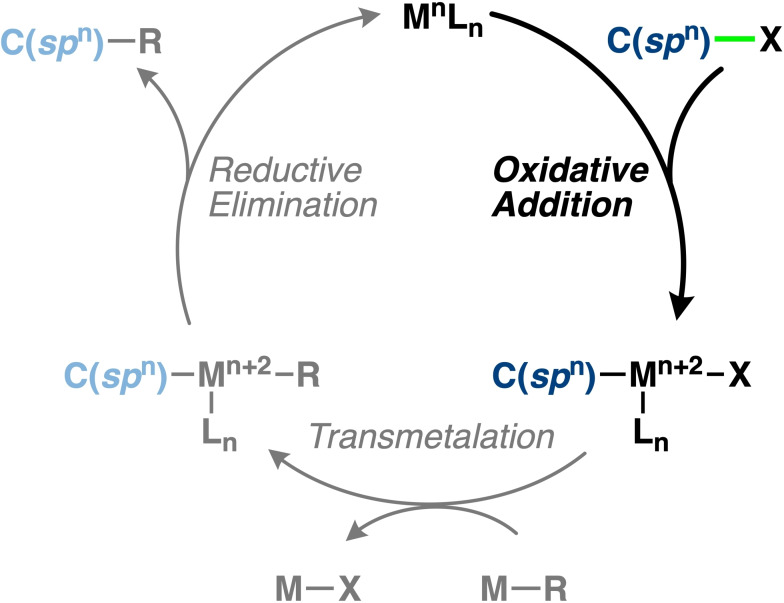
General transition‐metal‐catalyzed cross‐coupling mechanism.

Catalytic processes play an indispensable role in the chemist's toolbox and can offer exceptional control over the reactivity and selectivity of chemical transformation.^[1]^ The transition‐metal‐catalyzed cross‐coupling reaction (Scheme 1) offers a convenient strategy to forge C−C bonds.^[2]^ The oxidative addition step in the catalytic cycle plays an important role in the reactivity and selectivity, and several experimental and theoretical studies have found the oxidative addition step to be the rate‐determining step.^[3]^ What is currently needed is a quantitative model to rationalize the effect of the C(*sp* ^n^)−X hybridization of the substrate on the reactivity of the oxidative addition.^[4]^

**Scheme 2:**
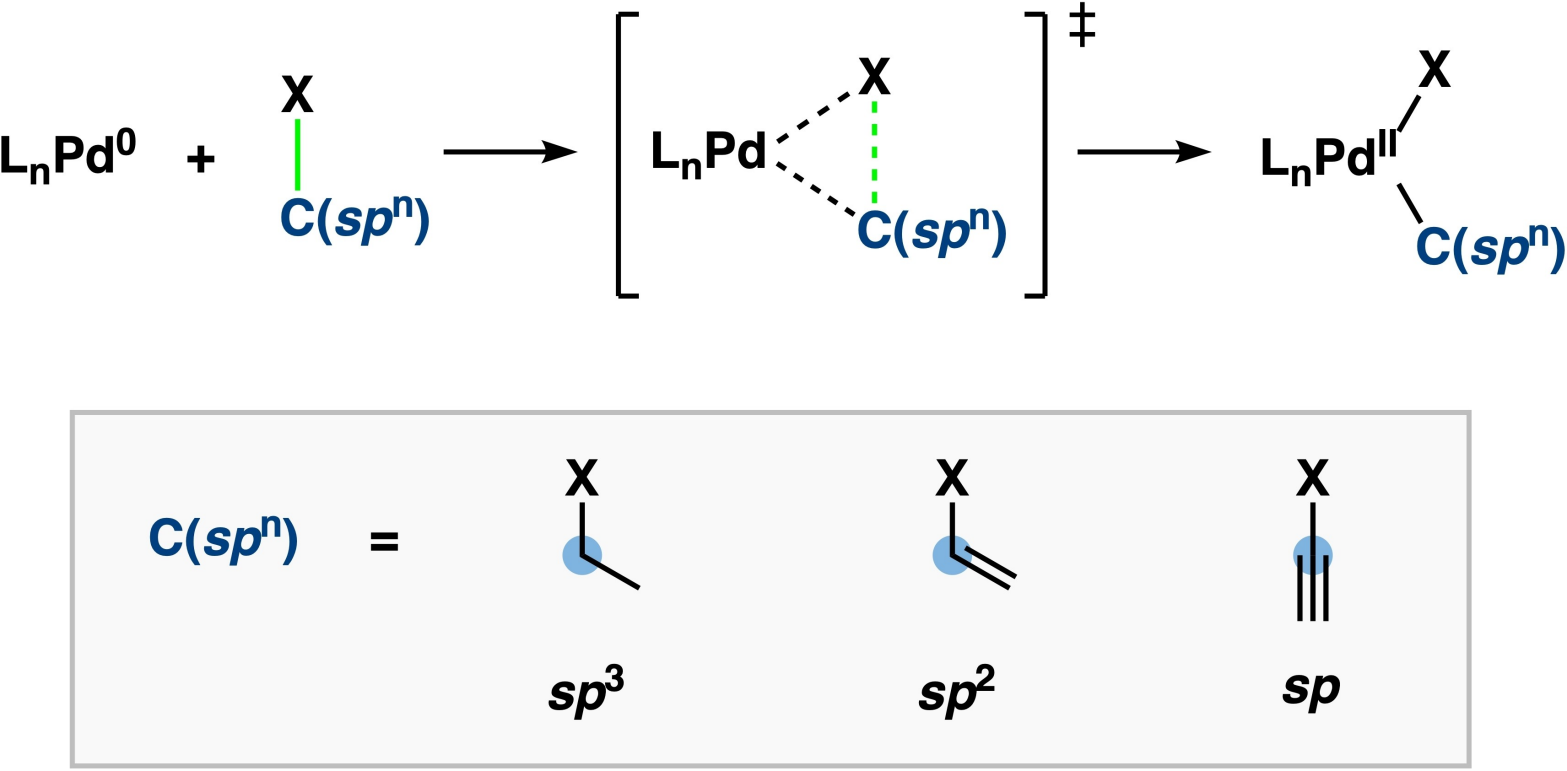
Model R−X bond activation reactions: R = H_3_C−CH_2_−, H_2_C=CH−, HC≡C−; X = H, CH_3_, Cl; PdL_n_ = Pd, PdCl^−^, Pd(PH_3_)_2_.

Thus, to unravel the effect of hybridization of C(*sp* ^n^)−X (n = 1–3) in the bond activation process, we have computationally explored the potential energy surface (PES) of the oxidative addition reaction of C−X (H, CH_3_, Cl) catalyzed by PdL_n_, i.e., PdL_n_ = Pd, PdCl^−^, and Pd(PH_3_)_2_, using relativistic density functional theory at ZORA‐BLYP/TZ2P (Scheme 2). We have chosen to represent common motifs of reactants in transition‐metal‐catalyzed reactions through archetypal model R−X substrates, with R = H_3_C−CH_2_−, H_2_C=CH−, HC≡C−. The activation strain model (ASM)^5^ and Kohn‐Sham molecular orbital (KS‐MO)^[6a]^ theory in combination with the energy decomposition analysis (EDA)[^6b^, ^6c^] have been used to unravel the reactivity trends and provide quantitative insights into the effect of hybridization of the carbon atom on the C(*sp* ^n^)−X bond activation process. This computational methodology provides deep physical insight into the factors controlling reactivity and has proven useful for the understanding, among others, of various types of organic and inorganic reactions.^[7]^

### Method

#### Computational details

All calculations were performed using the Amsterdam Density Functional (ADF2019.302) software package.^[8]^ The generalized gradient approximation (GGA) exchange‐correlation functional BLYP was used for the optimizations of all stationary points as well as the analyses.^[9]^ This level of theory has been widely tested with several ab initio reference benchmarks up until the coupled cluster CCSD(T).[^4h^, ^4i^, ^4j^] Scalar relativistic effects are taken into account using the zeroth‐order regular approximation (ZORA).^[10]^ The all‐electron TZ2P basis set has been employed, which is of triple‐ζ quality for all atoms and includes two polarization functions per element: 2p and 3d on H, 3d, and 4 f on C, P, Cl, and 5p and 4 f on Pd.^[11]^ For all computations, the accuracies of the fit scheme (Zlm fit) and the integration grid (Becke grid) were set to VERYGOOD, and no symmetry constraints were enforced. All calculated stationary points have been verified, by performing a vibrational analysis calculation,^[12]^ to be energy minima (no imaginary frequencies) or transition states (only one imaginary frequency). The potential energy surfaces of the oxidative addition reactions were obtained by performing intrinsic reaction coordinate (IRC) calculations,^[13]^ and then analyzed using the PyFrag 2019 program.^[14]^ All the structures were illustrated using CYLview.^[15]^

### Activation strain and energy decomposition analysis

To analyze and compare the reaction pathways of the different systems, we used the activation strain model (ASM) of chemical reactivity.^[5]^ This model is a fragment‐based approach in which the PES can be described and understood with respect to the reactants. It takes into account the rigidity of the reactants and the amount of deformation that occurs during the reaction, as well as their inclination to interact with each other during the oxidative addition. Using this model, the total energy, Δ*E*(ζ), is decomposed into the strain and interaction energy, Δ*E*_strain_(ζ) and Δ*E*_int_(ζ), respectively, and these values are then projected onto the reaction coordinate ζ as in the [Eq. 1].

(1) $$\Delta E\left(\zeta \right)=\Delta E{}_{\mathrm{strain}}\left(\zeta \right)+\Delta E{}_{\mathrm{int}}\left(\zeta \right)$$

In this equation, the energy required to deform the reactants from their equilibrium structure to the geometry they adopt during the reaction at an arbitrary point ζ of the reaction coordinate is identified by the strain energy, Δ*E*_strain_(ζ). On the other hand, the deformed reactants interact along the reaction coordinate and this contribution is entirely accounted for by the interaction energy, Δ*E*_int_(ζ).

A further decomposition of the interaction energy by means of quantitative Kohn‐Sham molecular orbital (KS‐MO) theory together with a canonical energy decomposition analysis (EDA) is also possible.^[6]^ The EDA decomposes the Δ*E*_int_(ζ) into the following three energy terms shown in [Eq. 2]

(2) $$\Delta E{}_{\mathrm{int}}\left(\zeta \right)=\Delta V{}_{\mathrm{elstat}}\left(\zeta \right)+\Delta E{}_{\mathrm{Pauli}}\left(\zeta \right)+\Delta E{}_{\mathrm{oi}}\left(\zeta \right)$$

From this equation, the quasi‐classical electrostatic potential interaction between the unperturbed charge distributions of the deformed reactants is accounted for by the Δ*V*_elstat_(ζ). This term is usually attractive. The Pauli repulsion, Δ*E*_Pauli_(ζ), on the other hand, considers the destabilizing interaction between the occupied orbitals of both fragments and it arises from the antisymmetrization of the wavefunction. Lastly, the orbital interaction energy, Δ*E*_oi_(ζ), accounts for polarization and charge transfer (e.g., HOMO–LUMO interactions) between the fragments.

In the present paper, the activation strain and energy decomposition diagrams are plotted using the PES from the intrinsic reaction coordinate (IRC) calculation. The energy is projected onto the bond stretch of the activated R⋅⋅⋅X bond. This is the dominant reaction coordinate for the studied oxidative addition reactions going from the reactant complex via the transition state to the product.^[4]^ For this reason, it is shown to be a reliable reaction coordinate for studying bond activation reactions. Consistent geometries were extracted from the IRC. To ensure that our consistent geometries were not skewed by the step size of the IRC, identical structures for all considered systems, in terms of the reaction coordinate, were obtained by linear interpolation of two adjacent IRC points.

### Thermochemistry

The bond dissociation energies (BDE), also known as bond enthalpies (Δ*H*_BDE_), are calculated at normal temperature and pressure (NTP, i.e., 298.15 K and 1 atm) from electronic bond energies (Δ*E*) and vibrational frequencies using the canonical thermochemistry relations for an ideal gas [Eq. 3].^[16]^

(3) $$\Delta H{}_{\mathrm{BDE}}=\Delta E+\Delta E{}_{\mathrm{trans},298.15}+\Delta E{}_{\mathrm{rot},298.15}+\Delta E{}_{\mathrm{vib},0}+\Delta \left(\Delta E{}_{\mathrm{vib},0}\right){}_{298.15}$$

Herein, Δ*E*_trans,298.15_, Δ*E*_rot,298.15_, and Δ*E*_vib,0_ are the differences in translational, rotational, and zero‐point vibrational energy between the R−X substrate and the homolytically dissociated fragments, R^•^ and X^•^ radical. The last term, Δ(Δ*E*_vib,0_)_298.15_, is the vibrational correction energy to bring the system from 0 K to 298.15 K.

## Results and Discussion

### Reactants and reaction profiles

**Figure 1:**
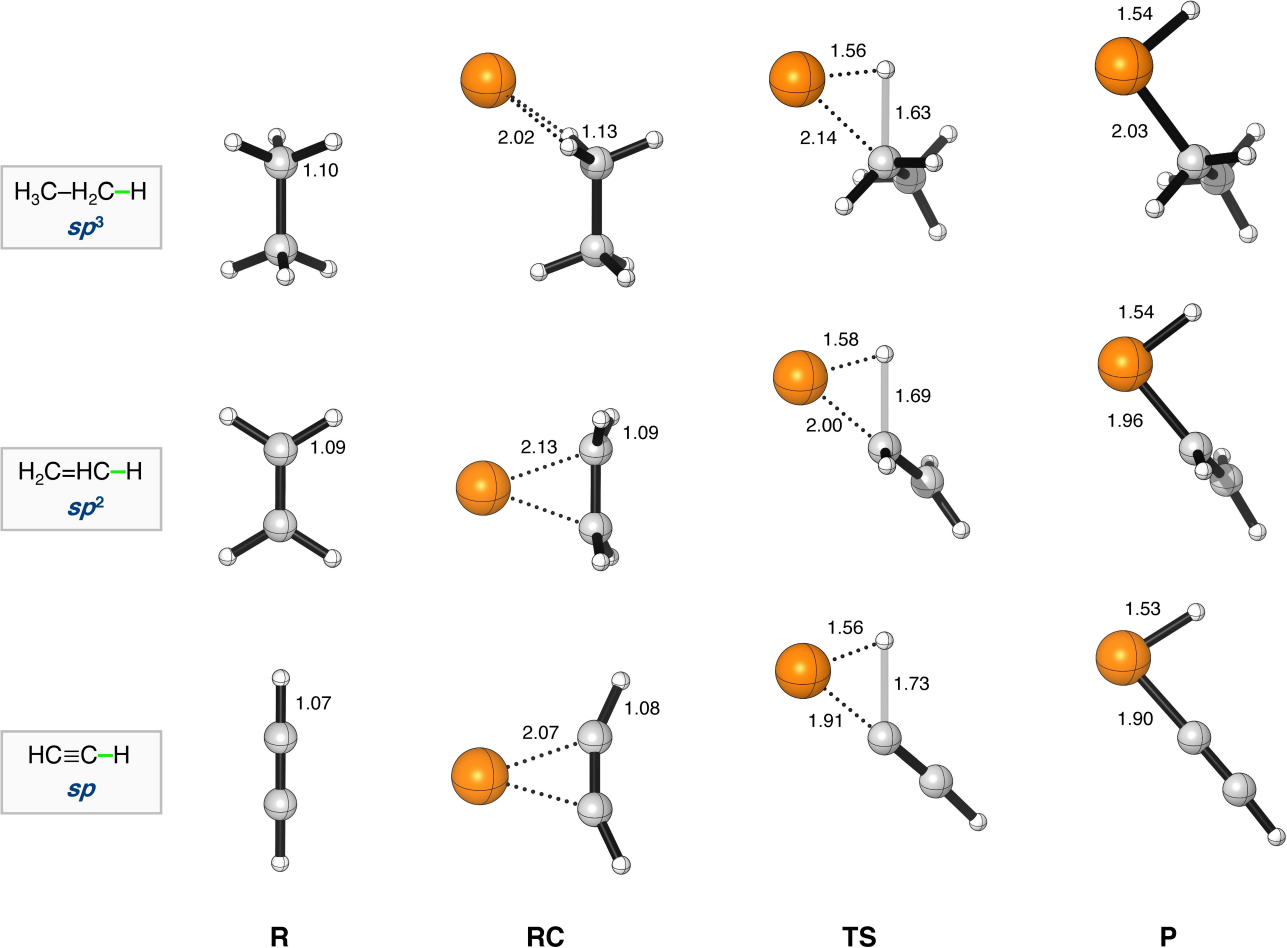
Stationary‐point structures (in Å) in the C−H bond activation of Pd+R−H (R = H_3_C−CH_2_−, H_2_C=CH−, HC≡C−), computed at ZORA‐BLYP/TZ2P (C=gray, H=white, Pd=orange).

**Figure 2:**
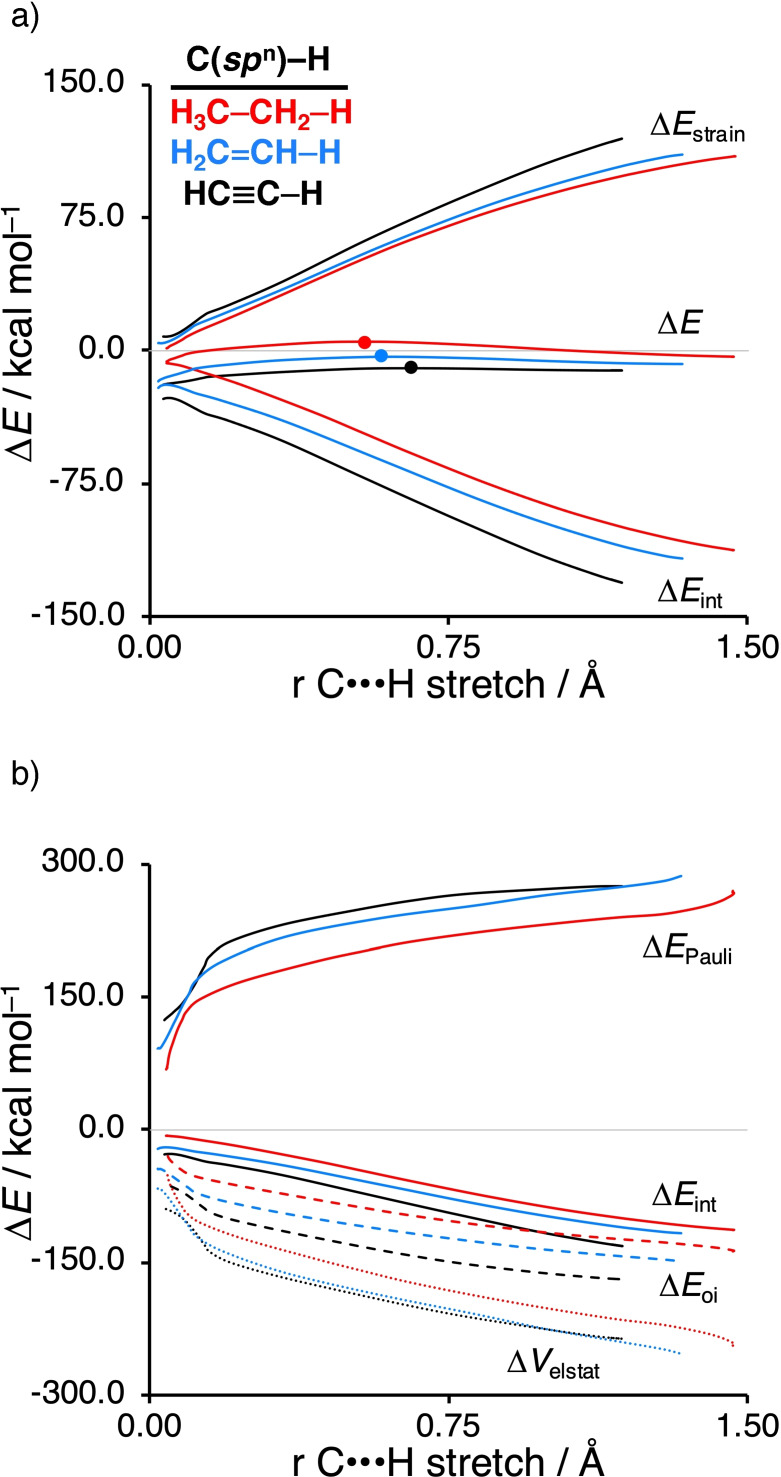
(a) Activation strain analysis and (b) energy decomposition analysis for the C−H activation of Pd+R−H with R = H_3_C−CH_2_− (red), H_2_C=CH− (blue), and HC≡C− (black), along the IRC projected on the C⋅⋅⋅H bond stretch. Computed at ZORA‐BLYP/TZ2P.

The results of our ZORA‐BLYP/TZ2P exploration are collected in Tables 1 and 2 and Figures 1 and 2. Full details and additional data can be found in Tables S1‐S9 and Figures S1‐S4 in the Supporting Information. Firstly, we have studied the bond strength (i.e., Δ*H*_BDE_) of the C(*sp* ^n^)−X bond of the substrate, which is an important aspect of the overall bond activation process (see below). We found, in line with experimental work,^[17]^ that the strength of the activated bond becomes substantially stronger following C(*sp* ^3^)−X, to C(*sp* ^2^)−X, to C(*sp*)−X. Along the same series, the activated C(*sp* ^n^)−X bond also becomes systematically shorter, going from 1.098 Å to 1.067 Å for X = H. These trends are found for each element X in our investigation, i.e., for C(*sp* ^n^)−H, C(*sp* ^n^)−C, as well as, C(*sp* ^n^)−Cl bonds.

**Table 1:** C(*sp* ^n^)−X (n = 1–3) bond lengths (in Å) and homolytic dissociation enthalpies (in kcal mol^−1^).^[a]^

Substrate	C(*sp* ^n^)−X (Å)	Δ*H*_BDE_
H_3_C−CH_2_−H	1.098	97.1
H_2_C=CH−H	1.089	106.0
HC≡C−H	1.067	131.1
H_3_C−CH_2_−CH_3_	1.541	79.8
H_2_C=CH−CH_3_	1.508	92.3
HC≡C−CH_3_	1.462	120.7
H_3_C−CH_2_−Cl	1.846	76.9
H_2_C=CH−Cl	1.764	87.8
HC≡C−Cl	1.652	106.2

[a] Computed at ZORA‐BLYP/TZ2P (enthalpies at 298.15 K).

**Table 2:** Energies relative to reactants (in kcal mol^−1^) of the stationary points along the C(*sp* ^n^)−X bond activation by PdL_n_ model catalysts.^[a]^

Activation Bond	Pd‐catalyst	Substrate	Bond type	RC	TS	P
C−H	Pd	H_3_C−CH_2_−H	*sp* ^3^−H	−6.7	4.6	−3.7
		H_2_C=CH−H	*sp* ^2^−H	−33.6	−3.5	−7.5
		HC≡C−H	*sp*−H	−33.5	−9.9	−11.2
	PdCl^−^	H_3_C−CH_2_−H	*sp* ^3^−H	−12.3	−4.2	−7.6
		H_2_C=CH−H	*sp* ^2^−H	−40.6	−16.4	−17.2
		HC≡C−H	*sp*−H	−41.9	−32.0	−33.6
	Pd(PH_3_)_2_	H_3_C−CH_2_−H	*sp* ^3^−H	^[b]^	34.3	29.5
		H_2_C=CH−H	*sp* ^2^−H	^[b]^	25.9	23.0
		HC≡C−H	*sp*−H	^[b]^	14.1	10.1
C−C	Pd	H_3_C−CH_2_−CH_3_	*sp* ^3^−CH_3_	−6.8	20.1	−8.2
		H_2_C=CH−CH_3_	*sp* ^2^ *−C*H_3_	−31.3	9.9	−8.4
		HC≡C−CH_3_	*sp−C*H_3_	−31.6	6.7	−9.2
	PdCl^−^	H_3_C−CH_2_−CH_3_	*sp* ^3^−CH_3_	−12.6	17.1	−8.7
		H_2_C=CH−CH_3_	*sp* ^2^ *−C*H_3_	−37.9	1.5	−9.5
		HC≡C−CH_3_	*sp−C*H_3_	−38.3	−7.2	−26.1
	Pd(PH_3_)_2_	H_3_C−CH_2_−CH_3_	*sp* ^3^−CH_3_	^[b]^	53.2	29.5
		H_2_C=CH−CH_3_	*sp* ^2^ *−C*H_3_	^[b]^	42.9	27.2
		HC≡C−CH_3_	*sp−C*H_3_	^[b]^	38.9	18.6
C−Cl	Pd	H_3_C−CH_2_−Cl	*sp* ^3^−Cl	−13.5	−0.9	−32.5
		H_2_C=CH−Cl	*sp* ^2^ *−C*l	−31.8	−15.9	−33.3
		HC≡C−Cl	*sp−C*l	−35.2	−17.5	−35.1
	PdCl^−^	H_3_C−CH_2_−Cl	*sp* ^3^−Cl	−17.8	−6.5	−53.1
		H_2_C=CH−Cl	*sp* ^2^ *−C*l	−45.4	−24.4	−55.4
		HC≡C−Cl	*sp−C*l	−49.7	−29.7	−65.7
	Pd(PH_3_)_2_	H_3_C−CH_2_−Cl	*sp* ^3^−Cl	^[b]^	27.2	−7.9
		H_2_C=CH−Cl	*sp* ^2^−Cl	^[b]^	15.4	−10.1
		HC≡C−Cl	*sp*−Cl	^[b]^	12.6	−23.6

[a] Electronic energies computed at ZORA‐BLYP/TZ2P. [b] RC is unbound.

Recently, we showed that the underlying physical mechanism behind the strengthening and shortening of the C(*sp* ^n^)−X bond is the reduction in steric congestions, i.e., the weakening in the destabilizing steric (Pauli) repulsion between the C(*sp* ^n^)‐group and the X‐atom as the number of substituents around the pertinent carbon atoms goes down from 4, to 3, to 2, along *sp* ^3^, to *sp* ^2^, to *sp* hybridization.^[18]^ In the present study, we also investigated the bond strength of *sp* ^2^_aryl_−X in C_6_H_5_−X (X = H, CH_3_, Cl) since arylic C−X bonds are also popular targets in experimental bond‐activation and cross‐coupling reaction studies. The bond strength of C(*sp* ^2^)_aryl_−X bonds is very similar to that of C(*sp* ^2^)−X in H_2_C=CH−H. For example, the Δ*H*_BDE_ of the C−H bond in benzene and ethene is 107.8 and 106.0 kcal mol^−1^, respectively (see Table S1).

The results of our computed ZORA‐BLYP/TZ2P reaction profiles for the studied C(*sp* ^n^)−X bond activation reactions are collected in Table 2 and Figure 1. Here we note that ZORA‐BLYP‐D3(BJ)/TZ2P yielded identical reactivity trends, however, systemically lower reaction barriers (see Table S2). The reactions generally proceed via a reactant complex (RC), and a transition state (TS), towards the product (P). Based on the reaction profiles, several clear trends emerged from the data. In the first place, the reaction barrier for the bond activation process decreased along C(*sp* ^3^)−X>C(*sp* ^2^)−X>C(*sp*)−X. For example, for L=no ligand and X = H, the barrier is going from +4.6, −3.5, −9.9 kcal mol^−1^ along this series. This trend is found for all bonds, C−H, C−C, and C−Cl, and for any model catalyst, Pd, PdCl^−^ and Pd(PH_3_)_2_. Note that in the gas phase, the effective reaction barrier is computed as the energy difference between the transition state and the separate reactants. The reaction barrier decreases going from C−C>C−H>C−Cl, in line with earlier work on C(*sp* ^3^)−X bonds.^4g^ Here, we find that the activation of C(*sp* ^2^)_aryl_−X closely follows the reactivity trends found for the *sp* ^2^ in H_2_C=CH−X. For example, the Δ*E* ^≠^ of the C−H activation of benzene and ethene is −1.4 and −3.5 kcal mol^−1^, respectively (see Table S3).

### Activation strain analyses

Next, we applied the activation strain model (ASM) of chemical reactivity to gain quantitative insight into the physical factors governing the bond activation process. Figure 2 shows the computed activation strain diagrams (ASDs) of the C−H activation between Pd+R−H with R = H_3_C−CH_2_− (red), H_2_C=CH− (blue), and HC≡C− (black). All other model reactions, i.e., C−C, C−Cl bonds; PdCl^−^, Pd(PH_3_)_2_ catalyst complexes, lead to similar ASDs with similar characteristics (see Figures S1 ‐ S3). In the ASM, the total energy profile (Δ*E*) is decomposed into the strain energy (Δ*E*_strain_) and the interaction energy (Δ*E*_int_): Δ*E*=Δ*E*_strain_ + Δ*E*_int_. The strain energy originates from the deformation of the reactants along the reaction coordinate. The interaction energy accounts for all chemical interactions between the deformed reactants. As found in Table 2, the reaction barrier decreases going from C(*sp* ^3^)−X>C(*sp* ^2^)−X>C(*sp*)−X, which can be traced back to the more stabilizing interaction energy between Pd and the substrate. The strain energy, on the other hand, gives rise to the opposite trend and becomes more destabilizing along this same series. This trend in strain energy originates from the increasingly stronger C−H bond (see Table 1), going from *sp* ^3^ (H_3_C−CH_2_−H: Δ*H*_BDE_=97.1 kcal mol^−1^), to *sp* ^2^ (H_2_C=CH−H: *H*_BDE_=106.0 kcal mol^−1^), to *sp* (HC≡C−H: Δ*H*_BDE_=131.1 kcal mol^−1^). However, the more significant strengthening in the stabilizing interaction energy between the catalyst and substrate along this series is able to overrule the increasingly destabilizing strain energy that goes with the activation of the relatively stronger bond. Additionally, again the data of the C(*sp* ^2^)_aryl_−X closely follow the reactivity trends found for the *sp* ^2^ (see Figure S4).

To understand the significantly more stabilizing interaction energy going from C(*sp* ^3^)−X to C(*sp* ^2^)−X to C(*sp*)−X, we apply a canonical energy decomposition analysis (EDA), in which we decompose the Δ*E*_int_ between the deformed reactants into three chemically intuitive energy terms, i.e., Δ*E*_Pauli_, Δ*V*_elstat_, and Δ*E*_oi_. The more stabilizing interaction along C(*sp* ^3^)−X, C(*sp* ^2^)−X, and C(*sp*)−X appears to be mainly caused by the more stabilizing orbital interactions. Note that these EDA plots should be interpreted with caution. We previously found,[^7c^, ^7d^, ^7e^] as one would expect, that the EDA results are strongly dependent on the distance between the reactants, in this case, the catalyst and substrate. The Pd⋅⋅⋅C distance is more than 0.2 Å longer for the Pd+H_3_C−CH_2_−H than Pd+HC≡C−H, i.e., 2.14 Å and 1.92 Å, respectively, at the same consistent TS‐like geometries (C⋅⋅⋅H bond stretch of 0.50 Å), as obtained from the IRC. The vastly different catalyst–substrate distance for C(*sp* ^3^)−X, C(*sp* ^2^)−X, and C(*sp*)−X is directly caused by the steric (Pauli) repulsion between the catalyst and substrate. The steric repulsion between the catalyst and substrate pushes the Pd substantially further away for H_3_C−CH_2_−H than HC≡C−H at the same C⋅⋅⋅H bond stretch. This effectively causes all EDA terms to be smaller in absolute magnitude for H_3_C−CH_2_−H (i.e., a less destabilizing Δ*E*_Pauli_, less stabilizing Δ*V*_elstat_ and Δ*E*_oi_).

To remedy the impact of the different catalyst–substrate bond lengths on the individual EDA terms, we artificially constrained the Pd⋅⋅⋅H and Pd⋅⋅⋅C bond lengths of Pd+H_3_C−CH_2_−H and Pd+H_2_C=CH−H to that of Pd+HC≡C−H, while keeping the C⋅⋅⋅H bond stretch constant. We constructed these geometries based on the above discussed consistent TS‐like geometries (C⋅⋅⋅H bond stretch of 0.50 Å) that we obtained from the IRC. As one would expect, the destabilizing steric (Pauli) repulsion significantly increases, i.e., ΔΔ*E*_Pauli_=+97.1 kcal mol^−1^, as does the orbital interaction, i.e., ΔΔ*E*_oi_=−27.0 kcal mol^−1^, and electrostatic attraction, i.e., ΔΔ*V*_elstat_=−63.5 kcal mol^−1^ for Pd+H_3_C−CH_2_–H.

Table 3 summarizes the data of this numerical experiment for Pd+R−H with R = H_3_C−CH_2_−, H_2_C=CH−, HC≡C−. The same trends are obtained for C−C and C−Cl bond activation (see Table S4 and S5). Table 3 shows that the catalyst–substrate interaction becomes more stabilizing going from C(*sp* ^3^)−X, to C(*sp* ^2^)−X, to C(*sp*)−X, in line with the ASM/EDA curves as function of the reaction coordinate in Figure 2. This trend originates from the steric (Pauli) repulsion, which becomes significantly less destabilizing along this series, i.e., 295.2, 277.1, and 245.3 kcal mol^−1^. The less destabilizing steric (Pauli) repulsion originates from the decreasing coordination number of the carbon atom in C(*sp* ^n^)−X along this series, causing less steric interactions between closed shells on the catalyst and closed shells with amplitude on the substituents of the substrate. In parallel, the initially found more stabilizing orbital interaction along the same series is maintained in our numerical experiment, however, this trend is significantly less steep than the steric (Pauli) repulsion, going only from −112.7, to −120.3, to −129.6 kcal mol^−1^. This can be directly traced back to the systematically lower‐lying σ*_C−X_ orbital of the substrate going from C(*sp* ^3^)−X, to C(*sp* ^2^)−X, to C(*sp*)−X. For example, along this series with X = H, the energy decreases from 1.2 (H_3_C−CH_2_−H), to 0.9 (H_2_C=CH−H), to 0.5 eV (HC≡C−H). The other substrates, i.e., C−C, C−Cl bonds also show the same behavior (see Table S6). The stabilization of the σ*_C−X_ orbital along this series is due to a decrease of antibonding overlap of the carbon 2*s* atomic orbital with the decreasing number of substituent lobes and an increase in bonding overlap of the carbon 2*p* atomic orbital with an increasingly more favorably aligned substituent lobe going from *sp* ^3^ to *sp* ^2^ to *sp*, respectively.^[18]^ This facilitates a stronger π‐backdonation from the catalyst to the substrate. Concluding, both the enhanced orbital interaction and less destabilizing steric (Pauli) repulsion cause a lower reaction barrier following C(*sp* ^3^)−X>C(*sp* ^2^)−X>C(*sp*)−X, in which the latter one is considerably more important.

**Table 3:** Activation strain and energy decomposition analyses (in kcal mol^−1^) for the C−H bond activation between Pd+R−H (R = H_3_C−CH_2_–, H_2_C=CH−, HC≡C−).^[a]^

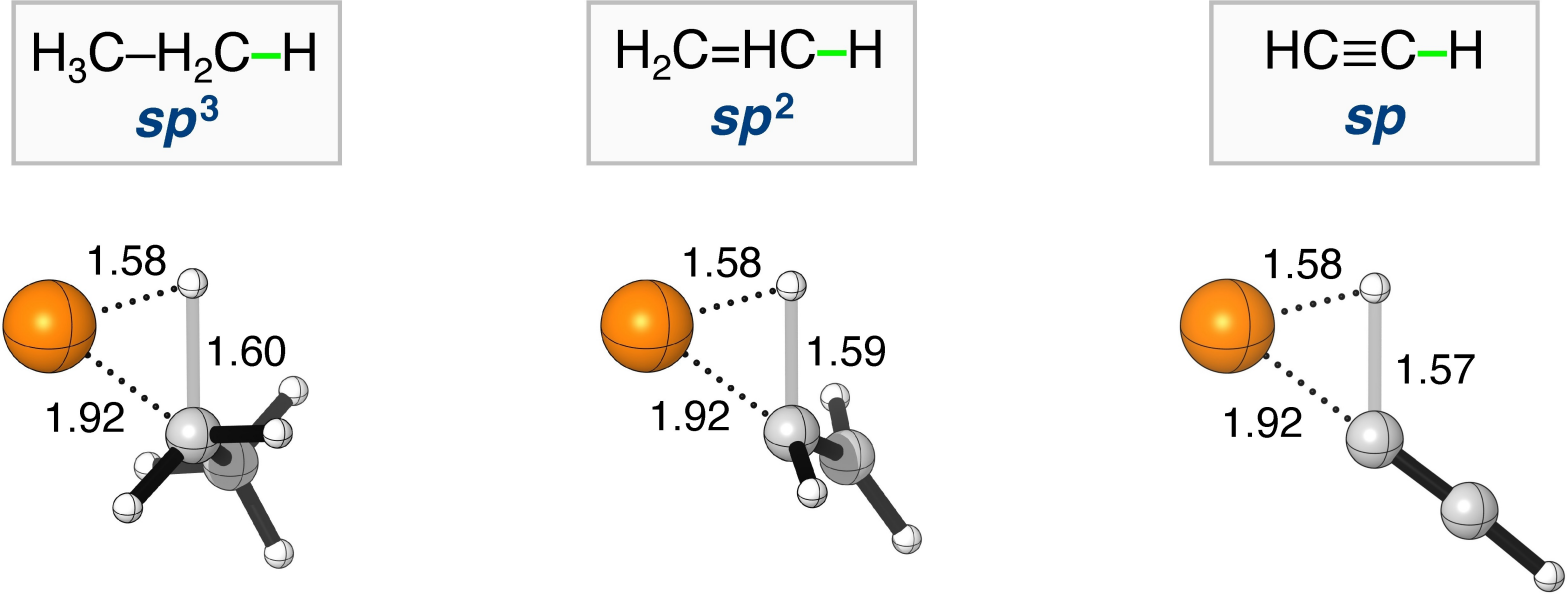						
Substrate	ΔE*	Δ*E*_strain_	Δ*E*_int_	Δ*V*_elstat_	Δ*E*_Pauli_	Δ*E*_oi_
H_3_C−CH_2_−H	11.2	48.2	−37.0	−219.6	295.3	−112.7
H_2_C=CH−H	−2.4	51.0	−53.4	−210.2	277.1	−120.3
HC≡C−H	−10.4	57.1	−67.5	−183.2	245.3	−129.6

[a] Numerical experiment at consistent TS‐like geometries (i.e., ΔE*) obtained from the IRC with a C⋅⋅⋅H bond stretch of 0.50 Å, a Pd⋅⋅⋅H distance of 1.58 Å, and a Pd⋅⋅⋅C distance of 1.92 Å (found on the PES for acetylene). Computed at ZORA‐BLYP/TZ2P.

## Conclusions

The reaction barrier for C(*sp* ^n^)−X bond activation (n = 1–3) systematically decreases along the series C(*sp* ^3^)−X>C(*sp* ^2^)−X>C(*sp*)−X, as follows from our quantum chemical analyses based on relativistic DFT. This reactivity trend is found for all bond types (C−H, C−C, C−Cl) and model catalysts (Pd, PdCl^−^, Pd(PH_3_)_2_) in this work. Interestingly, we establish that the trend of decreasing reaction barriers occurs whereas, along C(*sp* ^3^)−X, to C(*sp* ^2^)−X, to C(*sp*)−X, the bonds become substantially stronger and require more energy to break during the oxidative addition step.

Our activation strain and energy decomposition analyses reveal that the decreased reaction barrier going from C(*sp* ^3^)−X, to C(*sp* ^2^)−X, to C(*sp*)−X is caused mainly by a reduction in steric (Pauli) repulsion between the catalyst and the substrate. This originates from the decreasing coordination number of the carbon atom in C(*sp* ^n^)−X of the substrate going from *sp* ^3^, to *sp* ^2^, to *sp*. This trend is somewhat reinforced by an increasingly stabilizing orbital interaction, which is the result of a smaller and more favorable catalyst–substrate HOMO–LUMO gap, as the orbital energy of the substrate σ*_C−X_ orbital drops along *sp* ^3^, *sp* ^2^ and *sp* (cf. Vermeeren et al.^18^). In contrast, the required strain energy becomes significantly more destabilizing along this same series, which originates directly from the intrinsically stronger bond strength. However, the reduction in steric (Pauli) repulsion can overcome the required intrinsic higher destabilizing strain.

In all, the higher reactivity of the studied oxidative addition reactions going from C(*sp* ^3^)−X, to C(*sp* ^2^)−X, to C(*sp*)−X is not controlled by the bond strength of the R−X bond. Instead, it originates from a relief in the steric (Pauli) repulsion found between the catalyst and the substrate. The diminished steric (Pauli) repulsion for C(*sp* ^2^)−X and C(*sp*)−X bonds makes them an ideal substrate for transition‐metal‐catalyzed cross‐coupling reactions.

## Supporting Information

Additional computational results; Cartesian coordinates, energies, and the number of imaginary frequencies of all stationary points.

## Conflict of interest

The authors declare no conflict of interest.

1

## Supporting information

As a service to our authors and readers, this journal provides supporting information supplied by the authors. Such materials are peer reviewed and may be re‐organized for online delivery, but are not copy‐edited or typeset. Technical support issues arising from supporting information (other than missing files) should be addressed to the authors.

Supporting Information

## Data Availability

The data that support the findings of this study are available in the supplementary material of this article.
