# Supplementary material for: C(sp n)−X (n=1–3) Bond Activation by Palladium
Source: Chemistry. 2022 Jan 31;28(26):e202103953. doi: 10.1002/chem.202103953 (PMC9306469; doi:10.1002/chem.202103953)
Supplement: Supplementary file 1 — Supporting Information [file CHEM-28-0-s001.pdf]

Chemistry–A European Journal

Supporting Information

C(sp^n)–X (n = 1–3) Bond Activation by Palladium

Thomas Hansen, Xiaobo Sun, Marco Dalla Tiezza, Willem-Jan van Zeist, Jordi Poater, Trevor A. Hamlin,* and F. M. Bickelhaupt*

Contents

Figure S1. Activation strain analysis; and energy decomposition analysis, where ΔE_{Pauli} = solid lines, ΔE_{oi} = dashed lines, ΔV_{elstat} = dotted lines, of the C–H activation between Pd (a,b), $PdCl^{-}$ (c,d), and $Pd(PH_3)_2$ (e,f) + R–H with R = $H_3C–CH_2$– (red), H_2C=CH– (blue), and HC≡C– (black), along the IRC projected on the C•••H bond stretch. Computed at ZORA-BLYP/TZ2P.

Figure S2. Activation strain analysis; and energy decomposition analysis, where ΔE_{Pauli} = solid lines, ΔE_{oi} = dashed lines, ΔV_{elstat} = dotted lines, of the C–H activation between Pd (a,b), $PdCl^{-}$ (c,d), and $Pd(PH_3)_2$ (e,f) + R–CH_3 with R = $H_3C–CH_2$– (red), H_2C=CH– (blue), and HC≡C– (black), along the IRC projected on the C•••C bond stretch. Computed at ZORA-BLYP/TZ2P.

Figure S3. Activation strain analysis; and energy decomposition analysis, where ΔE_{Pauli} = solid lines, ΔE_{oi} = dashed lines, ΔV_{elstat} = dotted lines, of the C–H activation between Pd (a,b) and $PdCl^{-}$ (c,d) and $Pd(PH_3)_2$ (e,f) + R–Cl with R = $H_3C–CH_2$– (red), H_2C=CH– (blue), and HC≡C– (black), along the IRC projected on the C•••Cl bond stretch. Computed at ZORA-BLYP/TZ2P.

Figure S4. Activation strain analysis; and energy decomposition analysis, where ΔE_{Pauli} = solid lines, ΔE_{oi} = dashed lines, ΔV_{elstat} = dotted lines, for Pd + H_2C=CH–Cl (blue) and C_6H_5–Cl (black) of the C–H (a,b), C–C (c,d) and C–Cl activation (e,f), along the IRC projected on the C•••Cl bond stretch. Computed at ZORA-BLYP/TZ2P.

Table S1. Bond lengths of the activated sp^3–H, sp^2_{aryl}–H, sp^2–H, and *sp*–H in Å and the corresponding homolytic bond dissociation enthalpies at 298 K.[a]

Table S2. Energies relative to reactants (in kcal mol^{-1}) of the stationary points of the C(sp^n)–X bond (n = 1–3) activation along the PES, computed with and without dispersion-corrected DFT.

Table S3. Energies relative to reactants (in kcal mol^{-1}) of the stationary points of the sp^3–H, sp^2_{aryl}–H, sp^2–H, and *sp*–H activation along the PES.[a]

Table S4. Activation strain and energy decomposition analyses (in kcal mol^{-1}) for the C–C bond activation between Pd + R–CH_3 (R = $H_3C–CH_2$–, H_2C=CH–, HC≡C–).[a]

Table S5. Activation strain and energy decomposition analyses (in kcal mol^{-1}) for the C–Cl bond activation between Pd + R–Cl (R = $H_3C–CH_2$–, H_2C=CH–, HC≡C–).[a]

Table S6. $\sigma^*_{C\text{–}X}$ orbital energies (in eV) for all studied substrates.[a]

Table S7. Cartesian coordinates (in Å), energies (*E*, *H* and *G*, in kcal mol^{-1}), and number of imaginary vibrational frequencies (N_{imag}) of all stationary points and transition states of C–H activation in the gas phase, computed at ZORA-BLYP/TZ2P.

Table S8. Cartesian coordinates (in Å), energies (*E*, *H* and *G*, in kcal mol^{-1}), and number of imaginary vibrational frequencies (N_{imag}) of all stationary points and transition states of C–C activation in the gas phase, computed at ZORA-BLYP/TZ2P.

Table S9. Cartesian coordinates (in Å), energies (*E*, *H* and *G*, in kcal mol^{-1}), and number of imaginary vibrational frequencies (N_{imag}) of all stationary points and transition states of C–Cl activation in the gas phase, computed at ZORA-BLYP/TZ2P.

Table S10. Cartesian coordinates (in Å), energies (*E*, *H* and *G*, in kcal mol^{-1}), and number of imaginary vibrational frequencies (N_{imag}) of all stationary points and transition states of the C–H, C–Cl and C–C bond activation in the gas phase, computed at ZORA-BLYP-D3(BJ)/TZ2P.

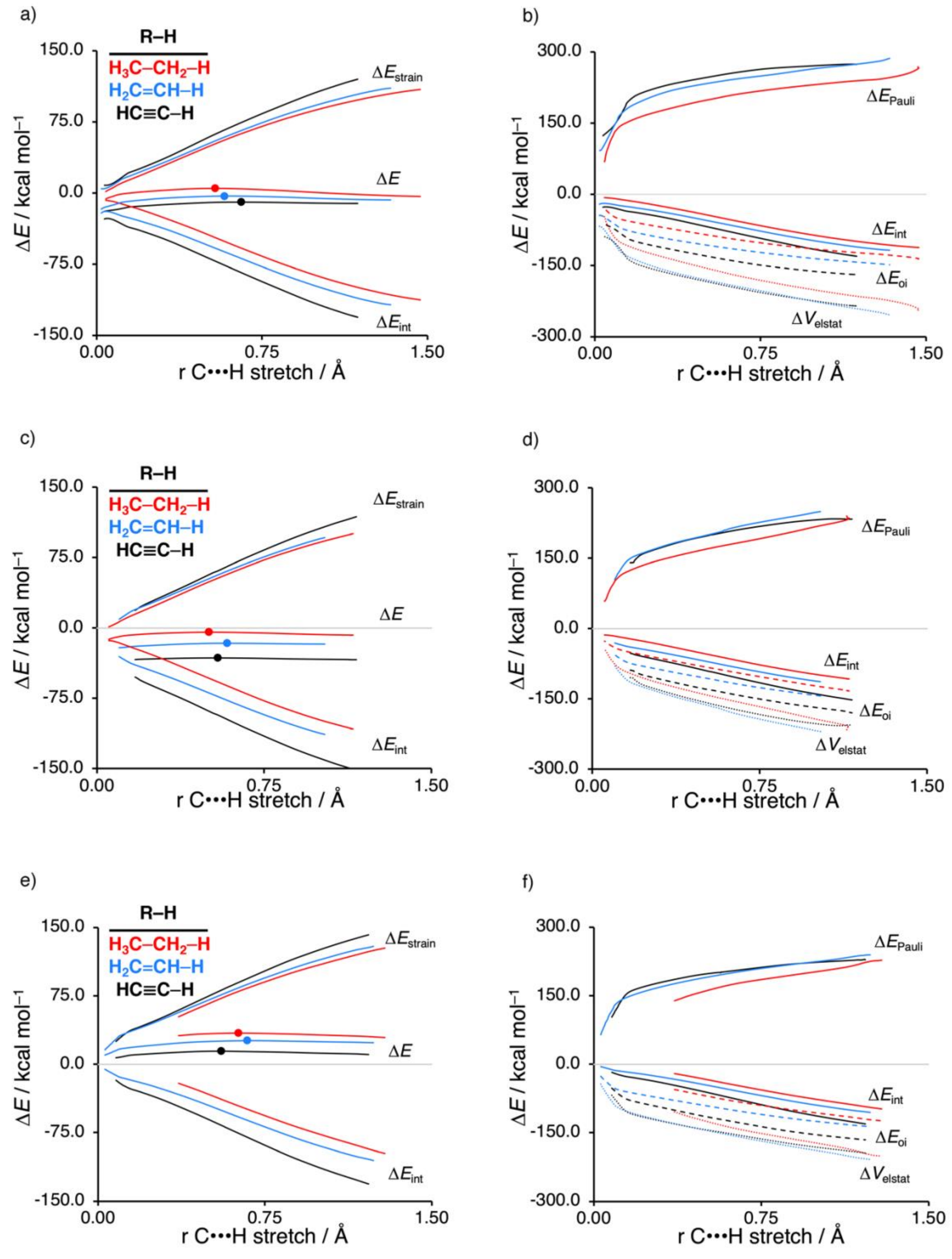

Figure S1. Activation strain analysis; and energy decomposition analysis, where $\Delta E_{\mathrm{Pauli}}$ = solid lines, ΔE_{oi} = dashed lines, $\Delta V_{\mathrm{elstat}}$ = dotted lines, of the C–H activation between Pd (a,b), $PdCl^{-}$ (c,d), and $Pd(PH_3)_2$ (e,f) + R–H with R = $H_3C–CH_2–$ (red), $H_2C=CH–$ (blue), and HC≡C– (black), along the IRC projected on the C•••H bond stretch. Computed at ZORA-BLYP/TZ2P.

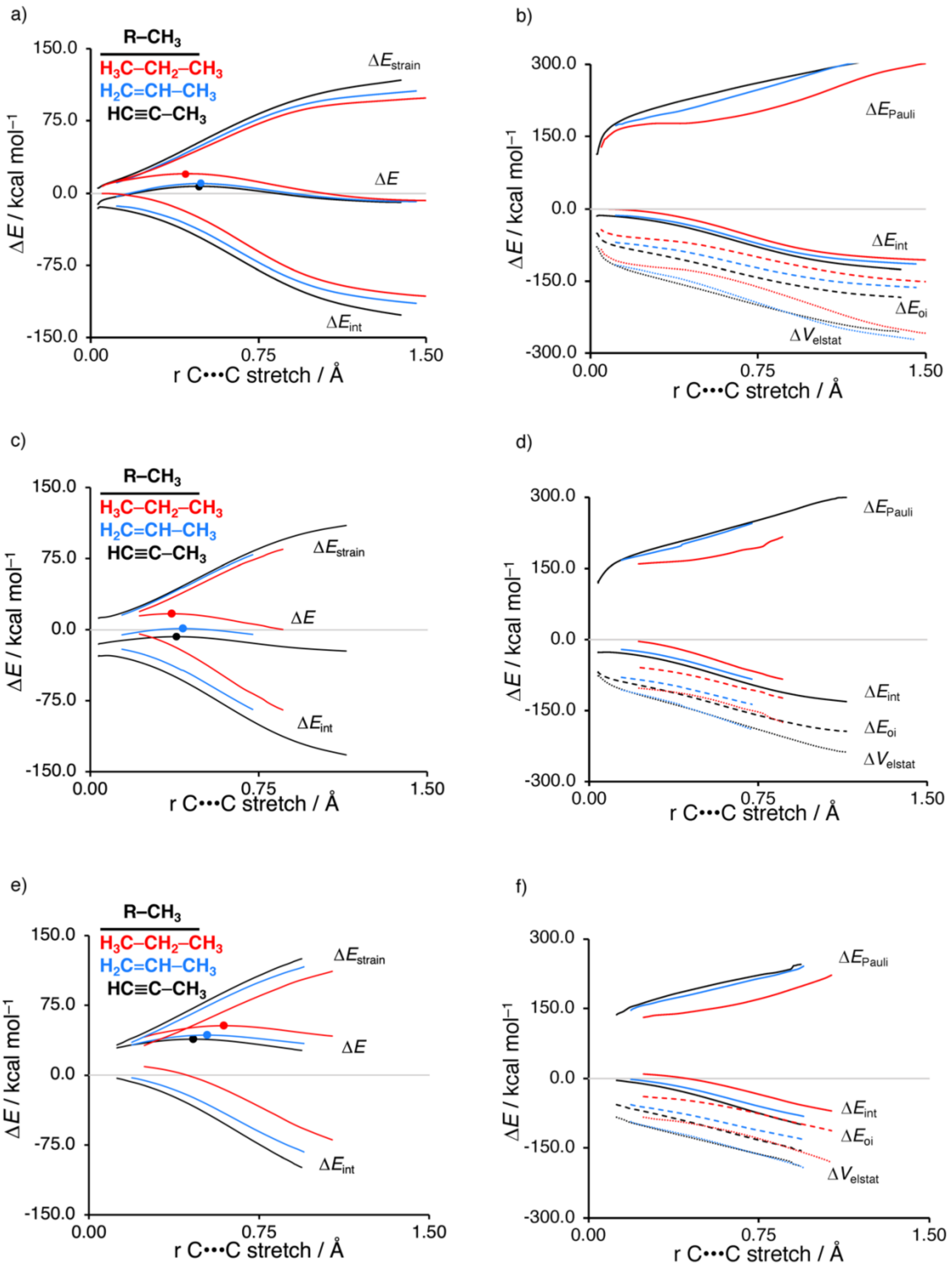

Figure S2. Activation strain analysis; and energy decomposition analysis, where $\Delta E_{\mathrm{Pauli}}$ = solid lines, ΔE_{oi} = dashed lines, $\Delta V_{\mathrm{elstat}}$ = dotted lines, of the C–H activation between Pd (a,b), $PdCl^{-}$ (c,d), and $Pd(PH_3)_2$ (e,f) + R–CH_3 with R = H_3C–CH_2– (red), H_2C=CH– (blue), and HC≡C– (black), along the IRC projected on the C•••C bond stretch. Computed at ZORA-BLYP/TZ2P.

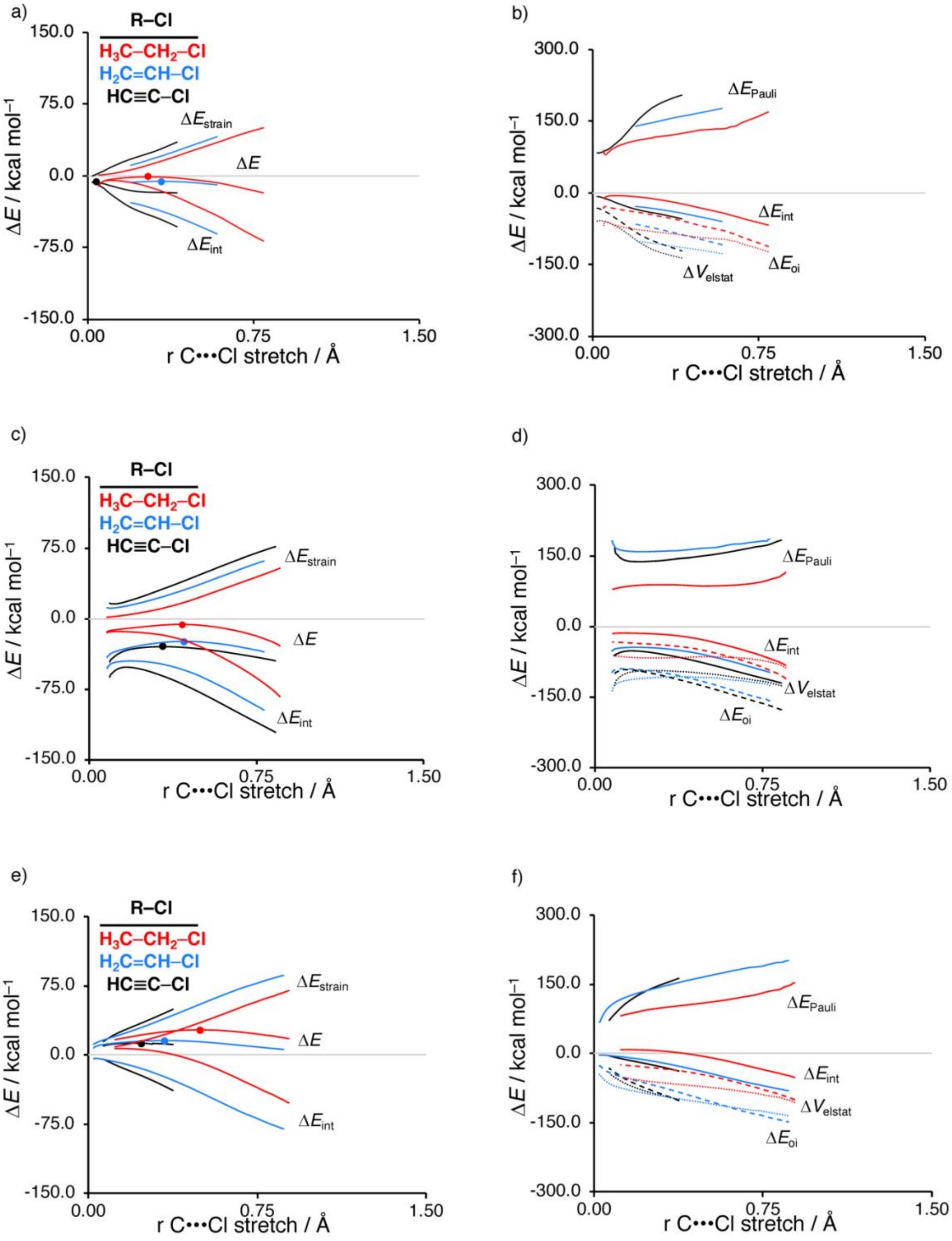

Figure S3. Activation strain analysis; and energy decomposition analysis, where ΔE_{Pauli} = solid lines, ΔE_{oi} = dashed lines, ΔV_{elstat} = dotted lines, of the C–H activation between Pd (a,b) and $PdCl^-$ (c,d) and $Pd(PH_3)_2$ (e,f) + R–Cl with R = $H_3C–CH_2$– (red), $H_2C=CH$– (blue), and HC≡C– (black), along the IRC projected on the C•••Cl bond stretch. Computed at ZORA-BLYP/TZ2P.

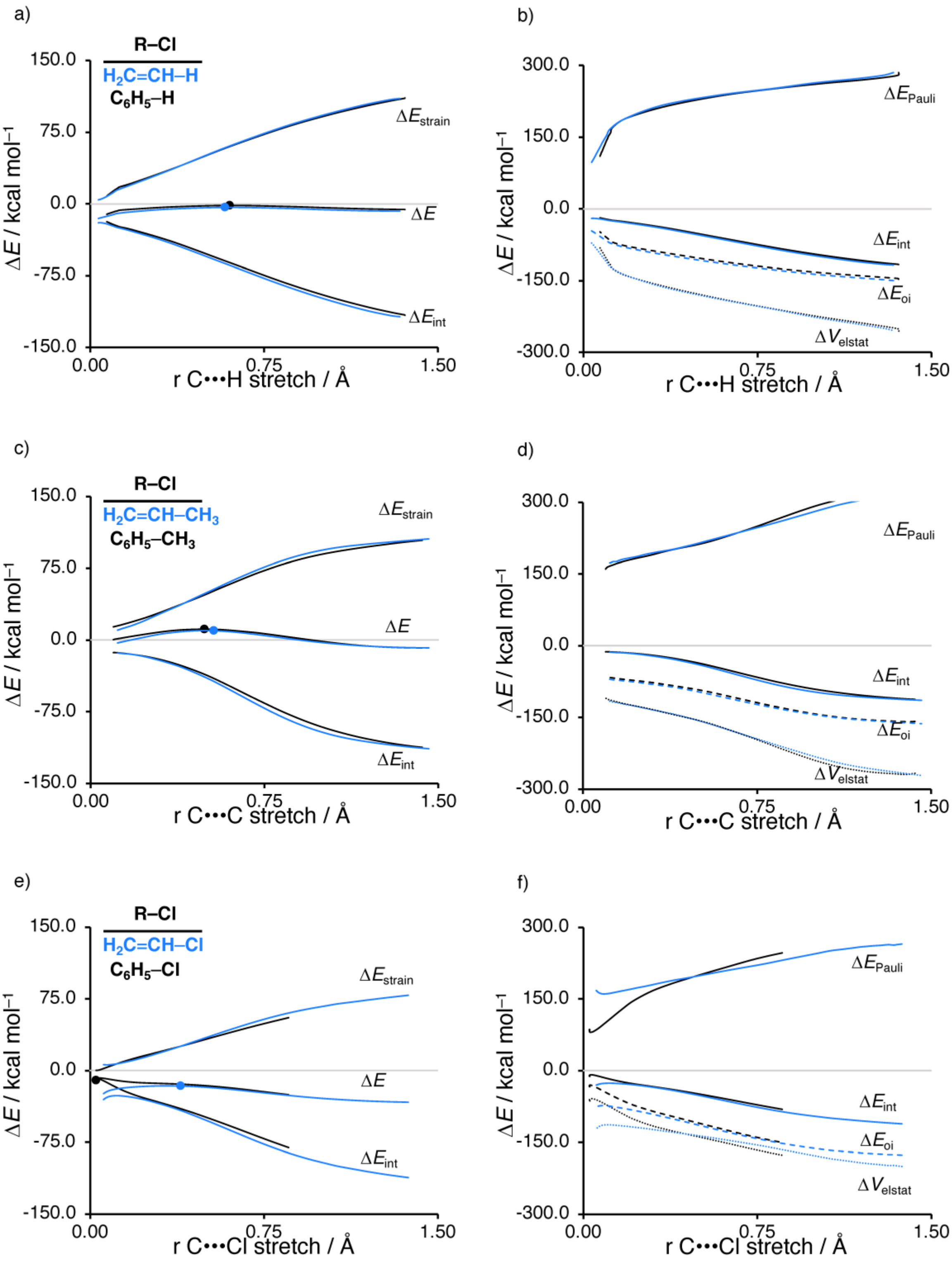

Figure S4. Activation strain analysis; and energy decomposition analysis, where $\Delta E_{\mathrm{Pauli}}$ = solid lines, ΔE_{oi} = dashed lines, $\Delta V_{\mathrm{elstat}}$ = dotted lines, for Pd + H_2C=CH–Cl (blue) and C_6H_5–Cl (black) of the C–H (a,b), C–C (c,d) and C–Cl activation (e,f), along the IRC projected on the C•••Cl bond stretch. Computed at ZORA-BLYP/TZ2P.

Table S1. Bond lengths of the activated sp^3–H, sp^2_{aryl}–H, sp^2–H, and sp–H in Å and the corresponding homolytic bond dissociation enthalpies at 298.15 K.[a]

Substrate	ΔH_{BDE}	C(sp^n)–X (Å)
H_3C–CH_2–H	97.1	1.098
C_6H_5–H	107.8	1.089
H_2C=CH–H	106.0	1.089
HC≡C–H	131.1	1.067
H_3C–CH_2–CH_3	79.8	1.541
C_6H_5–CH_3	93.3	1.517
H_2C=CH–CH_3	92.3	1.508
HC≡C–CH_3	120.7	1.462
H_3C–CH_2–Cl	76.9	1.846
C_6H_5–Cl	90.5	1.772
H_2C=CH–Cl	87.8	1.764
HC≡C–Cl	106.2	1.652

[a] Computed at ZORA-BLYP/TZ2P (kcal mol^{-1}).

Table S2. Energies relative to reactants (in kcal mol^{-1}) of the stationary points of the C(sp^n)–X bond (n = 1–3) activation along the PES, computed with and without dispersion-corrected DFT.

Activation	Pd-catalyst	Substrate	Bond type	RC	TS	P
C–H	Pd[a]	$H_3C–CH_2–H$	sp^3–H	–6.7	4.6	–3.7
		$H_2C=CH–H$	sp^2–H	–33.6	–3.5	–7.5
		HC≡C–H	*sp*–H	–33.5	–9.9	–11.2
	Pd[b]	$H_3C–CH_2–H$	sp^3–H	–10.9	0.3	–7.5
		$H_2C=CH–H$	sp^2–H	–37.3	–7.4	–11.3
		HC≡C–H	*sp*–H	–36.7	–13.0	–14.2
C–C	Pd[a]	$H_3C–CH_2–CH_3$	$sp^3–CH_3$	–6.8	20.1	–8.2
		$H_2C=CH–CH_3$	$sp^2–CH_3$	–31.3	9.9	–8.4
		$HC≡C–CH_3$	$sp–CH_3$	–31.6	6.7	–9.2
	Pd[b]	$H_3C–CH_2–CH_3$	$sp^3–CH_3$	–11.3	14.4	–13.0
		$H_2C=CH–CH_3$	$sp^2–CH_3$	–36.0	4.6	–12.8
		$HC≡C–CH_3$	$sp–CH_3$	–35.8	1.9	–13.3
C–Cl	Pd[a]	$H_3C–CH_2–Cl$	sp^3–Cl	–13.5	–0.9	–32.5
		$H_2C=CH–Cl$	sp^2–Cl	–31.8	–16.1	–33.3
		HC≡C–Cl	*sp*–Cl	–35.2	–18.4	–40.1
	Pd[b]	$H_3C–CH_2–Cl$	sp^3–Cl	–18.2	–5.8	–36.9
		$H_2C=CH–Cl$	sp^2–Cl	–36.3	–20.8	–37.3
		HC≡C–Cl	*sp*–Cl	–39.4	–22.6	–38.7

[a] Electronic energies computed at ZORA-BLYP/TZ2P. [b] Electronic energies computed at ZORA-BLYP-D3(BJ)/TZ2P.

Table S3. Energies relative to reactants (in kcal mol^{-1}) of the stationary points of the sp^3–H, sp^2_{aryl}–H, sp^2–H, and sp–H activation along the PES.[a]

Activation	Pd-catalyst	Substrate	Bond type	RC	TS	P
C–H	Pd	$H_3C–CH_2–H$	sp^3–H	–6.7	4.6	–3.7
		$C_6H_5–H$	sp^2_{aryl}–H	–21.5	–1.4	–5.6
		$H_2C=CH–H$	sp^2–H	–33.6	–3.5	–7.5
		$HC≡C–H$	sp–H	–33.5	–9.9	–11.2
	$PdCl^–$	$H_3C–CH_2–H$	sp^3–H	–12.3	–4.2	–7.6
		$C_6H_5–H$	sp^2_{aryl}–H	–27.0	–16.1	–17.6
		$H_2C=CH–H$	sp^2–H	–40.6	–16.4	–17.2
		$HC≡C–H$	sp–H	–41.9	–32.0	–33.6
	$Pd(PH_3)_2$	$H_3C–CH_2–H$	sp^3–H	[b]	34.3	29.5
		$C_6H_5–H$	sp^2_{aryl}–H	[b]	27.8	24.7
		$H_2C=CH–H$	sp^2–H	[b]	25.9	23.0
		$HC≡C–H$	sp–H	[b]	14.1	10.1
C–C	Pd	$H_3C–CH_2–CH_3$	sp^3–CH_3	–6.8	20.1	–8.2
		$C_6H_5–CH_3$	sp^2_{aryl}–H	–19.6	11.3	–8.1
		$H_2C=CH–CH_3$	sp^2–CH_3	–31.3	9.9	–8.4
		$HC≡C–CH_3$	sp–CH_3	–31.6	6.7	–9.2
	$PdCl^–$	$H_3C–CH_2–CH_3$	sp^3–CH_3	–12.6	17.1	–8.7
		$C_6H_5–CH_3$	sp^2_{aryl}–H	–28.5	1.6	–15.0
		$H_2C=CH–CH_3$	sp^2–CH_3	–37.9	1.5	–9.5
		$HC≡C–CH_3$	sp–CH_3	–38.3	–7.2	–26.1
	$Pd(PH_3)_2$	$H_3C–CH_2–CH_3$	sp^3–CH_3	[b]	53.2	29.5
		$C_6H_5–CH_3$	sp^2_{aryl}–H	[b]	46.0	27.7
		$H_2C=CH–CH_3$	sp^2–CH_3	[b]	42.9	27.2
		$HC≡C–CH_3$	sp–CH_3	[b]	38.9	18.6
C–Cl	Pd	$H_3C–CH_2–Cl$	sp^3–Cl	–13.5	–0.9	–32.5
		$C_6H_5–Cl$	sp^2_{aryl}–H	–11.5	–8.0	–33.3
		$H_2C=CH–Cl$	sp^2–Cl	–31.8	–16.1	–33.3
		$HC≡C–Cl$	sp–Cl	–35.2	–18.4	–40.1
	$PdCl^–$	$H_3C–CH_2–Cl$	sp^3–Cl	–17.8	–6.5	–53.1
		$C_6H_5–Cl$	sp^2_{aryl}–H	–20.3	–22.6	–55.5
		$H_2C=CH–Cl$	sp^2–Cl	–45.4	–25.6	–55.4
		$HC≡C–Cl$	sp–Cl	–49.7	–29.7	–65.7
	$Pd(PH_3)_2$	$H_3C–CH_2–Cl$	sp^3–Cl	[b]	27.2	–7.9
		$C_6H_5–Cl$	sp^2_{aryl}–H	[b]	19.5	–9.1
		$H_2C=CH–Cl$	sp^2–Cl	[b]	15.4	–10.1
		$HC≡C–Cl$	sp–Cl	[b]	12.6	–23.6

[a] Electronic energies computed at ZORA-BLYP/TZ2P. [b] Nonexistent: RC not stable.

Table S4. Activation strain and energy decomposition analyses (in kcal mol^{-1}) for the C–C bond activation between Pd + R–CH_3 (R = H_3C–CH_2–, H_2C=CH–, HC≡C–).[a]

Substrate	ΔE^*	ΔE_{strain}	ΔE_{int}	ΔV_{elstat}	ΔE_{Pauli}	ΔE_{oi}
H_3C–CH_2–CH_3	30.8	36.8	–6.0	–191.5	283.6	–98.1
H_2C=CH–CH_3	10.1	38.4	–28.3	–165.7	235.8	–98.4
HC≡C–CH_3	6.1	42.6	–36.4	–155.3	223.0	–104.2

[a] Analyses at consistent TS-like geometries (*i.e.*, ΔE^*) with a C•••H bond stretch of 0.40 Å a Pd•••C and Pd•••C bond distance of 2.21 Å and 1.94 Å, respectively. Computed at ZORA-BLYP/TZ2P.

Table S5. Activation strain and energy decomposition analyses (in kcal mol^{-1}) for the C–Cl bond activation between Pd + R–Cl (R = H_3C–CH_2–, H_2C=CH–, HC≡C–).[a]

Substrate	ΔE^*	ΔE_{strain}	ΔE_{int}	ΔV_{elstat}	ΔE_{Pauli}	ΔE_{oi}
H_3C–CH_2–Cl	26.0	6.5	19.5	–159.0	275.1	–96.6
H_2C=CH–Cl	–15.6	11.8	–27.4	–115.0	168.0	–80.4
HC≡C–Cl	–15.8	18.3	–34.2	–100.1	150.5	–84.6

[a] Analyses at consistent TS-like geometries (*i.e.*, ΔE^*) with a C•••H bond stretch of 0.20 Å a Pd•••Cl and Pd•••C bond distance of 2.06 Å and 2.45 Å, respectively. Computed at ZORA-BLYP/TZ2P.

Table S6. $\sigma^*_{C\text{–}X}$ orbital energies (in eV) for all studied substrates.[a]

Substrate	Bond type	$\sigma^*_{C\text{–}X}$
H_3C–CH_2–H	sp^3–H	1.2
H_2C=CH–H	sp^2–H	0.9
HC≡C–H	*sp*–H	0.5
H_3C–CH_2–CH_3	sp^3–CH_3	0.7
H_2C=CH–CH_3	sp^2–CH_3	0.3
HC≡C–CH_3	*sp*–CH_3	–0.2
H_3C–CH_2–Cl	sp^3–Cl	–0.1
H_2C=CH–Cl	sp^2–Cl	–0.3
HC≡C–Cl	*sp*–Cl	–0.7

[a] Computed at ZORA-BLYP/TZ2P.

Table S7. Cartesian coordinates (in Å), energies (*E*, *H* and *G*, in kcal mol^{-1}), and number of imaginary vibrational frequencies (N_{imag}) of all stationary points and transition states of the C–H activation in the gas phase, computed at ZORA-BLYP/TZ2P.

R: $PdCl^-$
E = -119.60
H = -116.86
G = -134.51
N_{imag} = 0

Pd	0.0000000	0.00000000	-0.00847700
Cl	0.0000000	0.00000000	2.32586400

R: $Pd(PH_3)_2$
E = -756.19
H = -718.30
G = -743.99
N_{imag} = 0

Pd	0.0000000	0.00000000	0.00000000
P	0.0000000	0.00000000	2.28490600
H	0.6092810	1.05530600	3.01943100
H	0.6092810	-1.05530600	3.01943100
H	-1.2185620	0.00000000	3.01943100
P	0.0000000	0.00000000	-2.28490600
H	-1.2185620	0.00000000	-3.01943100
H	0.6092810	-1.05530600	-3.01943100
H	0.6092810	1.05530600	-3.01943100

R: $H_3C–CH_2–H$
E = -896.24
H = -847.97
G = -864.88
N_{imag} = 0

C	0.00000000	0.00000000	0.76969800
H	0.00000000	1.02232700	1.16809400
H	0.88536100	-0.51116400	1.16809400
H	-0.88536100	-0.51116400	1.16809400
C	0.00000000	0.00000000	-0.76969800
H	-0.88536100	0.51116400	-1.16809400
H	0.88536100	0.51116400	-1.16809400
H	0.00000000	-1.02232700	-1.16809400

R: $C_6H_5–H$
E = -1666.47
H = -1601.88
G = -1621.74
N_{imag} = 0

C	1.21287600	-0.70025400	0.00000000
C	1.21287600	0.70025400	0.00000000
C	0.00000000	1.40050900	0.00000000
C	-1.21287600	0.70025400	0.00000000
C	-1.21287600	-0.70025400	0.00000000

```
C   0.00000000  -1.40050900   0.00000000
H   2.15553100   1.24449600   0.00000000
H   0.00000000   2.48899300   0.00000000
H  -2.15553100   1.24449600   0.00000000
H  -2.15553100  -1.24449600   0.00000000
H   0.00000000  -2.48899300   0.00000000
H   2.15553100  -1.24449600   0.00000000
```

R: $H_2C{=}CH{-}H$

E = -708.49
H = -674.86
G = -690.48
$\boldsymbol{N_{imag}}$ = 0

```
C   0.00000000   0.00000000   0.66671100
C   0.00000000   0.00000000  -0.66671100
H   0.00000000   0.92571500  -1.23922400
H   0.00000000   0.92571500   1.23922400
H   0.00000000  -0.92571500   1.23922400
H   0.00000000  -0.92571500  -1.23922400
```

R: HC≡C-H

E = -508.72
H = -489.97
G = -504.28
$\boldsymbol{N_{imag}}$ = 0

```
C   0.00000000   0.00000000  -0.60220200
C   0.00000000   0.00000000   0.60220200
H   0.00000000   0.00000000  -1.66958400
H   0.00000000   0.00000000   1.66958400
```

RC: Pd + $H_3C{-}CH_2{-}H$

E = -902.91
H = -853.86
G = -876.73
$\boldsymbol{N_{imag}}$ = 0

```
C   0.21141800  -2.77562100   0.00000000
C   0.87834200  -1.38802600   0.00000000
H   0.50508700  -3.35077200  -0.88699400
H  -0.88045300  -2.69131200   0.00000000
H   0.50508700  -3.35077200   0.88699400
H   1.97160300  -1.44694700   0.00000000
H   0.60143600  -0.82809900  -0.93567800
H   0.60143600  -0.82809900   0.93567800
Pd -0.21920300   0.76691900   0.00000000
```

TS: Pd + $H_3C{-}CH_2{-}H$

E = -891.60
H = -845.46
G = -867.18
$\boldsymbol{N_{imag}}$ = 1, -785.42 cm^{-1}

```
C   1.30496600  -0.64963400  -0.22874100
```

C	2.43449400	0.35104200	0.03892200
H	2.20224800	1.34601700	-0.35392000
H	2.64323200	0.45531000	1.10920000
H	3.35932000	0.00606000	-0.44984500
H	1.08209600	-0.75192000	-1.30306900
H	1.54453500	-1.64908900	0.14628300
H	0.31045200	-0.52922400	1.06239000
Pd	-0.69910600	0.05084000	0.01893100

P: Pd + $H_3C–CH_2–H$

E = -899.97

H = -853.20

G = -875.26

N_{imag} = 0

C	1.22590200	-0.62704200	-0.33034600
C	2.33420800	0.34561900	0.05722700
H	2.19636400	1.33661400	-0.38800900
H	2.40518500	0.46684900	1.14267400
H	3.30457100	-0.04221500	-0.29735300
H	1.14675500	-0.75997400	-1.42455300
H	1.33926400	-1.60617800	0.14659100
H	-0.42267800	-0.03917100	1.54412200
Pd	-0.68189200	-0.02657800	0.02364000

RC: Pd + C_6H_5–H

E = -1688.01

H = -1622.25

G = -1647.09

C	-0.34082600	-0.08064600	1.82129400
C	-1.62786000	0.37773500	1.36806500
C	-2.53216600	-0.56091200	0.79002600
C	-2.16650400	-1.89458800	0.64131800
C	-0.90362200	-2.34479500	1.08699700
C	-0.00891700	-1.46016700	1.67978600
H	-1.81034100	1.44318600	1.22960800
H	-3.49796200	-0.21312200	0.42874900
H	-2.85528500	-2.59625100	0.17486400
H	-0.62851100	-3.39015900	0.96104500
H	0.97060200	-1.80556500	2.00452500
H	0.45352200	0.63698100	2.02567600
Pd	-1.65253500	0.20786800	3.56396800

TS: Pd + C_6H_5–H

E = -1667.83

H = -1605.43

G = -1630.09

N_{imag} = 1, -690.03 cm^{-1}

C	0.09230400	-0.57852800	-0.21096600
C	-1.14588000	-1.25034500	-0.15418200
C	-1.18293400	-2.63773600	0.03385600
C	0.00558700	-3.37118800	0.13502500

C	1.23740200	-2.71221800	0.03924600
C	1.28654300	-1.32523400	-0.14883600
H	-2.07478800	-0.69460000	-0.25022700
H	-2.14469900	-3.14499700	0.09256100
H	-0.02793100	-4.45006600	0.27358000
H	2.16588300	-3.27762500	0.10224400
H	2.24827800	-0.82770200	-0.24070400
Pd	0.15447400	1.41983700	0.04467900
H	0.13275900	0.66961000	-1.34555700

P: Pd + C_6H_5-H

E = -1672.11
H = -1608.88
G = -1634.59

C	0.09266000	-0.56482800	-0.10678700
C	-1.14705500	-1.22622700	-0.08089200
C	-1.18314100	-2.62447200	0.02317300
C	0.00513200	-3.36224700	0.07633600
C	1.23714300	-2.70024800	0.02068200
C	1.28858700	-1.30251900	-0.08346500
H	-2.07687300	-0.66630900	-0.13848700
H	-2.14554700	-3.13305100	0.05412600
H	-0.02874200	-4.44749100	0.14946000
H	2.16586100	-3.26811600	0.04971800
H	2.25154000	-0.80197600	-0.14302500
Pd	0.15430100	1.40254900	0.02851600
H	0.15517200	1.42540400	-1.51833400

RC: Pd + H_2C=CH-H

E = -742.09
H = -706.90
G = -727.12
N_{imag} = 0

C	1.67933100	1.10374200	1.50540200
C	1.75116900	0.91997900	0.11178100
H	1.93110900	1.76703100	-0.54959400
H	1.80204200	2.09539100	1.93995600
H	1.85928600	0.26881200	2.18201400
H	1.98818800	-0.05897200	-0.30407800
Pd	-0.29532100	0.93501900	0.71296900

TS: Pd + H_2C=CH-H

E = -711.96
H = -680.47
G = -701.07
N_{imag} = 1, -700.17 cm^{-1}

C	1.27794500	-0.59099600	-0.13824500
C	2.31535600	0.24647200	0.01701800
H	0.18461100	-0.51081900	1.14094300
Pd	-0.65965500	-0.10322700	-0.12771500
H	2.21214400	1.25195700	0.41674000

H   3.32117900 -0.06444500 -0.27378200
H   1.43861700 -1.61984700 -0.46790800

P: Pd + H_2C=CH-H

E = -715.99
H = -684.42
G = -704.86
N_{imag} = 0

C   1.28060400 -0.56334200 -0.12746400
C   2.32130300   0.26638800 -0.00670500
H -0.57351300 -0.51042200   1.44191300
Pd -0.59252500 -0.00134500 -0.01674600
H   2.23119800   1.30173600   0.31377000
H   3.33033200 -0.08412500 -0.24095400
H   1.39394000 -1.61940700 -0.38433800

RC: Pd + HC≡C-H

E = -542.17
H = -522.25
G = -542.26
N_{imag} = 0

C   1.94141900   1.09693100   1.38489900
C   2.00682900   0.93282100   0.13744300
Pd 0.00148500   1.04125600   0.65374300
H   2.33848500   1.21312600   2.37887100
H   2.50525700   0.81045800 -0.80901000

TS: Pd + HC≡C-H

E = -518.61
H = -501.66
G = -521.86
N_{imag} = 1, -582.96 cm^{-1}

C   0.36657300   0.34522200   0.00000000
C   1.39779800   1.00106200   0.00000000
H   0.13883000 -1.36726400   0.00000000
Pd -1.25259500 -0.67231800   0.00000000
H   2.25665900   1.63596900   0.00000000

P: Pd + HC≡C-H

E = -519.91
H = -501.93
G = -522.47
N_{imag} = 0

C   0.37924800   0.34911700   0.00000000
C   1.39816100   1.01968200   0.00000000
H -0.19189500 -1.80976600   0.00000000
Pd -1.21863900 -0.68161600   0.00000000
H   2.25968900   1.65089500   0.00000000

RC: PdCl⁻ + H_3C-CH_2-H

E = -1028.11
H = -977.67
G = -1003.73
N_{imag} = 0

C	-0.97307500	-1.25125600	0.00000000
C	-0.06272600	-0.01048100	0.00000000
Pd	2.32727300	-0.00273000	0.00000000
H	-1.62341800	-1.27517400	0.88807300
H	-0.37438300	-2.16907400	0.00000000
H	-1.62341800	-1.27517400	-0.88807300
H	-0.63860000	0.92287400	0.00000000
H	0.58389600	0.00582900	0.92633300
H	0.58389600	0.00582900	-0.92633300
Cl	4.68032600	-0.00776100	0.00000000

TS: PdCl⁻ + H_3C-CH_2-H

E = -1020.06
H = -972.31
G = -997.63
N_{imag} = 1, -649.43 cm^{-1}

C	-0.10931400	-0.20280800	-2.06634900
Pd	-0.12648300	-0.15780700	0.08180500
C	1.24926200	-0.15746000	-2.79173400
H	1.93608500	0.54379600	-2.30358700
H	1.74142700	-1.13960700	-2.79560800
H	1.13481200	0.16052600	-3.84479600
H	-0.58185500	0.79322600	-2.06368100
H	-0.79767600	-0.87415000	-2.59854100
H	0.08610300	-1.32030000	-0.93442600
Cl	-0.27079300	0.64709700	2.32794400

P: PdCl⁻ + H_3C-CH_2-H

E = -1023.49
H = -975.11
G = -1001.38
N_{imag} = 0

C	-0.09876300	0.01812300	-0.20596000
Pd	1.53905900	0.19162800	-1.45708300
C	-0.38053000	-1.38823100	0.35246800
H	-0.37579400	-2.14824100	-0.43941900
H	0.37571300	-1.68635200	1.08961600
H	-1.36916500	-1.43792800	0.85066200
H	-0.89261100	0.31689200	-0.91284100
H	-0.09267700	0.75261800	0.61105200
H	2.10953500	0.18830300	-0.01939600
Cl	2.37790800	0.34055600	-3.73334300

RC: PdCl$^-$ + C_6H_5-H

E = -1813.05
H = -1745.79
G = -1774.87
N_{imag} = 0

C	-0.11081200	-0.16166500	2.38793600
C	-0.28368500	0.64418300	1.26331700
C	-1.01924300	0.17366900	0.16005300
C	-1.57606900	-1.10443600	0.18076900
C	-1.38123700	-1.95089100	1.30049300
C	-0.64521100	-1.47191700	2.41637300
H	0.14888300	1.64215300	1.23959700
H	-1.15430500	0.80961300	-0.71219800
H	-2.13628200	-1.47262200	-0.67560300
H	-1.66634600	-2.99963000	1.24025500
H	-0.37295900	-2.14758800	3.22418100
H	0.45965000	0.20102200	3.23998700
Pd	-2.84028753	-1.33546683	2.81278138
Cl	-4.77438623	-1.07736181	4.09530586

TS: PdCl$^-$ + C_6H_5-H

E = -1802.21
H = -1738.26
G = -1766.41
N_{imag} = 1, -571.76 cm^{-1}

C	0.10124000	-0.55869800	-0.12387100
C	-1.14285700	-1.24036300	-0.12124900
C	-1.21712900	-2.63435300	-0.00464100
C	-0.04979200	-3.40734500	0.07568700
C	1.19315500	-2.75984100	0.02726800
C	1.26717000	-1.36588400	-0.08950500
H	-2.06250300	-0.66258700	-0.19095800
H	-2.19378900	-3.12072100	0.02132000
H	-0.10738100	-4.49209400	0.16362700
H	2.11306700	-3.34484600	0.07854700
H	2.24330200	-0.88673400	-0.13419600
Pd	0.20245400	1.43270600	0.20750200
H	0.17496800	0.68740400	-1.17947500
Cl	0.30029500	3.46985000	1.43733900

P: PdCl$^-$ + C_6H_5-H

E = -1803.63
H = -1738.90
G = -1768.05
N_{imag} = 0

C	0.16156700	-0.55758600	0.13320800
C	-1.12340400	-1.11590400	-0.07515600
C	-1.30914600	-2.49496300	-0.25078500
C	-0.21167900	-3.36761200	-0.25129600
C	1.07380700	-2.83494000	-0.07888300
C	1.25633200	-1.45562000	0.09674000

H	-1.99375300	-0.46130000	-0.08746800
H	-2.31721400	-2.88928400	-0.39237700
H	-0.35442400	-4.43933500	-0.39013100
H	1.94185500	-3.49702900	-0.08489800
H	2.26705000	-1.06927000	0.22031600
Pd	0.39576200	1.35414200	0.67790800
H	0.50480500	1.31553000	-0.86177500
Cl	0.54788400	3.21601400	2.18791200

RC: $PdCl^-$ + $H_2C{=}CH{-}H$

E = -868.70
H = -832.11
G = -855.84
N_{imag} = 0

C	1.61284400	1.10312400	1.49931900
C	1.70498500	0.91986300	0.10342300
H	1.93155200	1.76261300	-0.55367300
H	1.76664100	2.09067400	1.94042600
H	1.79391000	0.27011400	2.18238800
H	1.95806800	-0.05808800	-0.31247400
Pd	-0.33816600	0.96683900	0.67514300
Cl	-2.71945600	0.92679700	0.51701500

TS: $PdCl^-$ + $H_2C{=}CH{-}H$

E = -844.51
H = -811.39
G = -835.62
N_{imag} = 1, -542.09 cm^{-1}

C	0.01557400	-0.24035100	-1.92714000
Pd	-0.14336800	-0.21504400	0.06562400
C	1.09517600	-0.17646000	-2.73755800
Cl	-0.46509000	0.73239200	2.23689500
H	0.10799200	-1.49369400	-0.80688800
H	0.99157400	0.06327300	-3.80111500
H	2.11176200	-0.32442500	-2.37453000
H	-0.96705200	-0.14341300	-2.40984400

P: $PdCl^-$ + $H_2C{=}CH{-}H$

E = -845.31
H = -811.52
G = -836.67
N_{imag} = 0

C	0.01490600	-0.22888200	-1.89524800
Pd	-0.12217600	-0.25684600	0.08199400
C	1.08498500	-0.19598800	-2.71455300
Cl	-0.51577000	0.75364900	2.24189000
H	0.22449000	-1.68925400	-0.38074000
H	0.97530600	0.00852900	-3.78574100
H	2.10511300	-0.34474600	-2.36058500
H	-0.97518200	-0.11638100	-2.36324900

RC: PdCl$^-$ + HC≡C-H

E = -670.19
H = -648.62
G = -672.18
***N*$_{imag}$** = 0

C	1.49929100	1.07434400	1.37253200
C	1.57078100	0.91192600	0.11704500
Pd	-0.42708100	0.98736600	0.63202500
Cl	-2.81407600	0.95714700	0.49808500
H	1.95504300	1.19608800	2.34342800
H	2.13530700	0.79281400	-0.79541200

TS: PdCl$^-$ + HC≡C-H

E = -660.33
H = -641.67
G = -665.31
***N*$_{imag}$** = 1, -543.88 cm^{-1}

C	1.97695000	0.13921600	0.00000000
Pd	0.05032300	-0.06512400	0.00000000
C	3.19940900	0.29368900	0.00000000
Cl	-2.30248200	0.26154400	0.00000000
H	1.10927100	-1.20611400	0.00000000
H	4.23807100	0.53842500	0.00000000

P: PdCl$^-$ + HC≡C-H

E = -661.95
H = -642.31
G = -666.34
***N*$_{imag}$** = 0

C	2.00398600	0.16365100	0.00000000
Pd	0.06663800	-0.01501500	0.00000000
C	3.22690600	0.29852800	0.00000000
Cl	-2.31341700	0.14045100	0.00000000
H	0.50882200	-1.47030600	0.00000000
H	4.27790300	0.48412300	0.00000000

RC: Pd(PH$_3$)$_2$ + H$_3$C–CH$_2$–H

E = -1652.27
H = -1566.71
G = -1600.44
***N*$_{imag}$** = 0

C	1.75414400	3.58897900	1.38218900
C	0.94937900	3.25246500	0.11461100
H	1.90757600	4.67073200	1.48453900
H	1.23838100	3.23987300	2.28546800
H	2.74364600	3.11503600	1.36341400
H	1.46337200	3.60483500	-0.78874400
H	-0.04120600	3.72434300	0.13623900
H	0.79911800	2.16989900	0.01269300
Pd	0.09473600	-1.13436200	-0.03090500
P	2.31813500	-1.65193500	-0.09187400

H	2.92704600	-2.35436100	0.98537900
H	3.33286600	-0.66008400	-0.20512000
H	2.82788900	-2.49340000	-1.11978300
P	-2.16097200	-0.78024800	0.02105200
H	-2.94214300	-1.24481400	1.11589600
H	-2.99564500	-1.32633100	-0.99368600
H	-2.72946700	0.52426900	-0.01455600

TS: $Pd(PH_3)_2$ + $H_3C{-}CH_2{-}H$

E = -1618.17
H = -1534.62
G = -1567.96
N_{imag} = 1, -712.74 cm^{-1}

Pd	-0.05959000	0.01651100	-0.03582200
H	2.03982600	2.65813100	0.25665000
H	1.86234600	-2.79084200	-0.29118800
H	0.01330500	-3.19060700	0.61768500
H	3.03914500	0.95608200	0.97875600
H	2.86761100	1.27473000	-1.09034700
H	0.12594300	-2.91861800	-1.46551700
P	2.00370400	1.24738600	0.04296300
P	0.54659700	-2.23350200	-0.29069800
C	-1.65312600	1.57451600	0.15992100
C	-1.85329600	1.99195300	1.62340400
H	-1.64540100	-0.14573600	-0.02300600
H	-2.48595900	2.89215100	1.68674300
H	-0.90548200	2.22053800	2.12457100
H	-2.34697300	1.20473900	2.20528100
H	-1.11789000	2.33581000	-0.41890700
H	-2.61997200	1.43951600	-0.33220800

P: $Pd(PH_3)_2$ + $H_3C{-}CH_2{-}H$

E = -1622.95
H = -1539.17
G = -1570.94
N_{imag} = 0

C	2.15734100	0.00020900	0.00000000
Pd	0.01684900	0.01670200	0.00000000
C	2.95606600	1.31112300	0.00000000
H	2.41076600	-0.59955900	-0.88652700
H	2.41076600	-0.59955900	0.88652700
H	0.23144800	1.59627900	0.00000000
H	-0.64864000	-3.09527700	-1.05548600
H	-3.40090900	-0.31057800	0.00000000
H	-2.73833400	1.39353700	-1.05774400
H	-0.64864000	-3.09527700	1.05548600
H	1.19109000	-3.09023100	0.00000000
H	-2.73833400	1.39353700	1.05774400
P	-0.03299400	-2.36527300	0.00000000
P	-2.27342200	0.56714000	0.00000000
H	4.04084800	1.10877700	0.00000000

H	2.73496200	1.92115500	0.88324500
H	2.73496200	1.92115500	-0.88324500

RC: Pd(PH$_3$)$_2$ + C$_6$H$_5$-H

E = -2423.06

H = -2321.81

G = -2355.53

N_{imag} = 0

C	-1.36885800	-2.04366900	-0.12638700
C	-0.27013600	-2.90322100	0.00090300
C	0.11563900	-3.71380600	-1.07452100
C	-0.59754800	-3.66555600	-2.27911700
C	-1.69636000	-2.80648200	-2.40755500
C	-2.08130700	-1.99622300	-1.33160200
H	0.28385500	-2.94074700	0.93735500
H	0.96970800	-4.38140200	-0.97438500
H	-0.29811200	-4.29539000	-3.11508300
H	-2.25084900	-2.76870900	-3.34373600
H	-2.93521400	-1.32837800	-1.43112700
H	-1.66917900	-1.41342000	0.71068600
H	-5.07789000	-1.70301600	3.02623500
H	-4.81888400	-1.04755000	5.01752000
H	-3.53896700	-2.52432600	4.21565200
Pd	-2.59505100	0.40497900	3.14505600
P	-1.18436600	2.07863100	2.48597200
H	0.21908000	1.86499900	2.39300600
H	-1.31995200	2.69941200	1.21310800
H	-1.09839000	3.28839700	3.22939800
P	-4.02057000	-1.23511700	3.85507900

TS: Pd(PH$_3$)$_2$ + C$_6$H$_5$-H

E = -2394.92

H = -2295.15

G = -2331.51

N_{imag} = 1, -683.38 cm^{-1}

N_{imag} =

C	-0.05442000	-0.50539500	-0.31750700
C	-1.20174500	-1.28583700	-0.07351000
C	-1.09189800	-2.64047700	0.26927400
C	0.16548800	-3.25059900	0.35103300
C	1.31174600	-2.49422200	0.07901300
C	1.20250700	-1.13954200	-0.26379900
H	-2.19117300	-0.83812200	-0.14744800
H	-1.99344500	-3.22018100	0.46469300
H	0.25017800	-4.30405800	0.61126100
H	2.29594900	-2.95922300	0.12529000
H	2.10688200	-0.57660000	-0.48763700
Pd	-0.19094600	1.60846400	-0.41901900
H	-0.22834800	0.59036200	-1.66273300
P	-0.03589200	2.12634400	1.92807100
H	-0.99910200	1.53877000	2.79522000

H   1.10242100   1.64613000   2.63420400
H -0.05346800   3.40937300   2.55425200
P -0.40683800   3.65649100 -1.55313600
H -1.56002000   3.86956800 -2.35838800
H -0.41362800   4.95729900 -0.96515900
H   0.53620100   3.97599700 -2.56901900

P: $Pd(PH_3)_2$ + C_6H_5-H

E = -2397.95
H = -2297.09
G = -2333.11
N_{imag} = 0

C -0.04385900 -0.45366800 -0.17140500
C -1.19026100 -1.24006000   0.05049400
C -1.08587100 -2.61246700   0.32411500
C   0.16899700 -3.23038500   0.37261300
C   1.31716300 -2.46389700   0.14233700
C   1.21042300 -1.09156600 -0.13092600
H -2.18088900 -0.78962600   0.00680500
H -1.98944300 -3.19779100   0.49311000
H   0.25056700 -4.29584600   0.58054000
H   2.30067200 -2.93246800   0.16868100
H   2.12063900 -0.52353000 -0.31809200
Pd -0.19627200   1.60569900 -0.49711200
H -0.28148900   1.06539800 -1.99639900
P -0.02897700   1.94884400   1.86565200
H -0.98909000   1.29665300   2.68298300
H   1.12120400   1.42767800   2.51425900
H -0.05144400   3.19704600   2.55430400
P -0.40736400   3.75134500 -1.42013300
H -1.55579000   4.01573200 -2.21183900
H -0.42643400   4.98598200 -0.70620200
H   0.55151700   4.15030000 -2.38809000

RC: $Pd(PH_3)_2$ + H_2C=CH-H

E = -1464.74
H = -1393.83
G = -1427.18
N_{imag} = 0

C   2.83546800   4.05246100 -1.49216400
C   2.05245300   3.55224300 -2.44944600
H -4.39196200 -2.70296400 -0.17965800
H -4.30017500 -0.90679600   0.92598600
H -4.17938100 -2.76540900   1.91956000
H   1.72237700 -0.85082900   0.36924600
H   1.61417300 -2.60638900 -0.79735100
P -3.55933000 -2.11706300   0.81476100
Pd -1.28657000 -2.08872800   0.58404100
P   0.98776500 -2.06940400   0.36119300
H   1.81889900 -2.74358800   1.29907200
H   2.50895600   4.10498600 -0.45478500

H	3.83421600	4.43001200	-1.70716400
H	1.05357800	3.17515600	-2.23454100
H	2.37870800	3.50053500	-3.48694600

TS: Pd(PH$_3$)$_2$ + H$_2$C=CH-H

E = -1438.78

H = -1370.50

G = -1401.17

N_{imag} = 1, -628.72 cm^{-1}

Pd	-0.06559300	-0.03520700	0.02133900
H	1.97061500	2.57286900	0.44463600
H	1.95846100	-2.75426700	-0.41178400
H	0.27448500	-3.20385600	0.76852900
H	3.19256300	0.85161400	0.53862000
H	2.55128500	1.58185100	-1.32850200
H	0.08047700	-3.02645400	-1.32460300
P	1.96693900	1.24798200	-0.07279000
P	0.62449800	-2.27336400	-0.24803300
C	-1.48361100	1.48104400	0.28087800
C	-1.93294700	2.02401800	1.42129000
H	-1.64589400	-0.26850300	0.18703700
H	-2.52379900	2.94312100	1.41294200
H	-1.73307500	1.59399100	2.40166200
H	-1.78123200	1.94087500	-0.66595900

P: Pd(PH$_3$)$_2$ + H$_2$C=CH-H

E = -1441.67

H = -1371.74

G = -1403.26

N_{imag} = 0

Pd	-0.17779000	-0.23741900	0.00362500
H	1.82749700	2.03097200	1.21910000
H	2.07338000	-2.75771900	-0.29962700
H	0.34515000	-3.36950200	0.75856800
H	3.09542300	0.81788100	0.01348800
H	1.79812200	2.23089800	-0.89348500
H	0.28079700	-3.16852800	-1.34957700
P	1.72139400	1.19681000	0.07577700
P	0.69337700	-2.40619900	-0.22468600
C	-1.37855200	1.44578000	0.15368500
C	-1.60428500	2.20037800	1.23650600
H	-1.62299200	-0.92236000	0.02171000
H	-2.24073300	3.08885000	1.18842300
H	-1.18830100	1.97406600	2.21869500
H	-1.86394800	1.72686200	-0.78595200

RC: Pd(PH_3)$_2$ + HC≡C-H

E = -1264.93
H = -1209.18
G = -1241.62
N_{imag} = 0

C	0.77891000	4.55989600	-3.01291700
C	1.53063200	5.29436000	-2.42384600
H	-3.44102800	-1.52632800	0.42482000
H	-2.77848500	-1.01928900	2.36325200
H	-3.22598700	-3.02764700	1.89177500
H	2.94654200	-1.93650000	0.67869700
H	2.29135100	-2.32753800	-1.28991300
P	-2.45210900	-1.96337000	1.34998000
Pd	-0.28942900	-2.29312800	0.69485900
P	1.87473700	-2.61954100	0.03898800
H	2.47106700	-3.91016900	0.09718800
H	0.11297200	3.90819800	-3.53392400
H	2.19607600	5.94541400	-1.90157900

TS: Pd(PH_3)$_2$ + HC≡C-H

E = -1250.84
H = -1196.93
G = -1227.35
N_{imag} = 1, -683.77 cm^{-1}

Pd	-0.05714100	0.02142700	-0.01537400
H	2.23461200	2.23539400	0.88499000
H	1.83262800	-2.76117600	0.06002200
H	-0.09798400	-3.14772000	0.82520100
H	3.35356700	0.76836300	-0.15241500
H	2.16967300	2.15197200	-1.22895400
H	0.20054800	-2.98912200	-1.26254800
P	2.01726400	1.26561000	-0.12944000
P	0.51220400	-2.24922900	-0.08986200
C	-1.38183200	1.52811400	0.08637300
C	-2.15401400	2.47426900	0.14669900
H	-1.65251100	-0.09053300	0.07959500
H	-2.78729100	3.33235300	0.19922000

P: Pd(PH_3)$_2$ + HC≡C-H

E = -1254.81
H = -1198.86
G = -1229.54
N_{imag} = 0

Pd	-0.16612100	-0.15096400	-0.04204200
H	1.91980000	2.15425900	1.02849700
H	1.97776100	-2.69085400	-0.16678700
H	0.22573500	-3.21362800	0.91870400
H	3.15623000	0.81869500	-0.08293000
H	1.88196500	2.19049800	-1.10276800
H	0.17888800	-3.14243600	-1.20668700
P	1.79642400	1.24528700	-0.05082900

P	0.60212900	-2.32925300	-0.12436400
C	-1.23752200	1.54481400	0.02258900
C	-1.84957600	2.60091600	0.05994700
H	-1.60057100	-0.81459200	-0.02969300
H	-2.39985900	3.51544500	0.09257500

Table S8. Cartesian coordinates (in Å), energies (*E*, *H* and *G*, in kcal mol^{-1}), and number of imaginary vibrational frequencies (N_{imag}) of all stationary points and transition states of the C–C activation in the gas phase, computed at ZORA-BLYP/TZ2P.

R: $H_3C–CH_2–CH_3$
E = -1258.69
H = -1192.20
G = -1211.83
N_{imag} = 0

C	0.03545700	0.03003800	0.77542100
H	0.11394300	1.06848500	1.12329000
H	0.90417200	-0.51769700	1.16046600
H	-0.86322000	-0.40501700	1.23225100
C	-0.03621200	-0.03614000	-0.76055300
C	-1.24306300	0.72037500	-1.34364200
H	0.89054400	0.37448200	-1.18582800
H	-0.07820500	-1.08780000	-1.07783000
H	-1.21171300	1.78383400	-1.07246800
H	-2.18798300	0.30970900	-0.96415200
H	-1.26661000	0.65546300	-2.43829100

R: $C_6H_5–CH_3$
E = -2030.91
H = -1948.50
G = -1972.09
N_{imag} = 0

C	-1.22826500	0.82967300	-0.03391100
C	-2.43476000	0.10946100	-0.07192300
C	-2.44140600	-1.28968700	-0.04544200
C	-1.23591300	-1.99807000	0.01788000
C	-0.02656700	-1.29446400	0.05224400
C	-0.02558900	0.10482900	0.02550500
H	-3.37932600	0.65003800	-0.12561200
H	-3.38818300	-1.82609100	-0.07813200
H	-1.23878200	-3.08611800	0.03552300
H	0.91766100	-1.83455300	0.09602100
H	0.92231900	0.64170800	0.04834000
C	-1.22582700	2.34633200	-0.03023000
H	-0.31099100	2.74642500	-0.48244200
H	-1.28186500	2.73748400	0.99582700
H	-2.08416900	2.74955300	-0.58006100

R: $H_2C=CH–CH_3$
E = -1074.51

H = -1022.82
G = -1041.75
N_{imag} = 0

C	-1.23082900	0.70440000	0.59750300
C	-1.16495000	0.47610500	-0.71713900
H	-1.19664900	1.32825200	-1.40046900
H	-1.31488000	1.71214200	0.99774800
H	-1.20309500	-0.11030100	1.32076500
C	-1.04745700	-0.87871900	-1.36308400
H	-0.13586500	-0.94605100	-1.97367700
H	-1.89166800	-1.06597700	-2.04188600
H	-1.02168500	-1.68107100	-0.61683000

R: HC≡C−CH_3
E = -878.89
H = -841.96
G = -860.30
N_{imag} = 0

C	0.00000000	0.00000000	1.01475400
C	0.00000000	0.00000000	-0.19291400
H	0.51199400	0.88680000	-2.04740300
H	-1.02398900	0.00000000	-2.04740300
H	0.51199400	-0.88680000	-2.04740300
C	0.00000000	0.00000000	-1.65258700
H	0.00000000	0.00000000	2.08117600

RC: Pd + H_3C−CH_2−CH_3
E = -1265.51
H = -1198.33
G = -1223.46
N_{imag} = 0

C	0.03246100	-0.05236700	-0.03517200
H	0.39776600	0.98233300	-0.01997800
C	-1.50877800	-0.04770700	0.01214700
C	0.59200300	-0.78208100	-1.27032100
H	0.41447400	-0.53080400	0.87827300
H	-1.92823200	-1.05980500	0.03858600
H	-1.86428500	0.46271000	0.94887200
H	-1.91506300	0.42998400	-0.92417500
H	0.25909100	-1.82808900	-1.29798600
H	0.25828300	-0.30066100	-2.19895900
H	1.68903200	-0.78073300	-1.26735600
Pd	-2.99775300	1.85442100	0.00577000

TS: Pd + H_3C−CH_2−CH_3
E = -1238.63
H = -1173.57
G = -1197.68
N_{imag} = 1, -475.13 cm^{-1}

C	0.04051200	-0.06590700	0.01655900
C	2.00643700	-0.01564100	0.10782400

H -0.03229700 -0.69775400   0.90183900
C -0.32745600 -0.81076600 -1.26886600
H -0.70003900   0.76439300   0.18824100
H   2.78482800   0.77110700 -0.08162300
H   2.17436700 -0.43010000   1.10149500
H   2.13819600 -0.77147100 -0.66525800
H -1.38107600 -1.12527900 -1.23546700
H -0.19947900 -0.17943800 -2.15462800
H   0.28138300 -1.71354500 -1.40575200
Pd 1.00642200   1.89640100 -0.01416600

P: Pd + $H_3C-CH_2-CH_3$

E = -1266.88
H = -1201.14
G = -1226.53
N_{imag} = 0

C   0.01867100 -0.01079800   0.01654600
C   3.10118300 -0.05932100 -0.08146700
H   0.07961300   0.53101500 -0.93365800
C -0.08676900   0.92438800   1.21721900
H -0.82050600 -0.72871300 -0.03011400
H   3.96453400 -0.69749300 -0.31969100
H   2.93710000   0.67500000 -0.87486700
H   3.22500900   0.42453700   0.89240400
H -1.00301400   1.53397600   1.13763400
H -0.13645100   0.37918900   2.16600300
H   0.76012800   1.61803100   1.26649400
Pd 1.54260300 -1.35571100   0.03179500

RC: Pd + $C_6H_5-CH_3$

E = -2050.48
H = -1966.93
G = -1993.96
N_{imag} = 0

C -1.76191500 -0.58056900   0.49910900
C -2.95570100 -1.35076200   0.31565500
C -2.92114100 -2.73864100   0.22593100
C -1.70173400 -3.43712300   0.35806600
C -0.52575000 -2.73679600   0.60405100
C -0.52835300 -1.31186500   0.67815000
H -3.89886400 -0.82246500   0.18169200
H -3.84358700 -3.28898400   0.05051200
H -1.68426000 -4.52183800   0.27611700
H   0.41979800 -3.26652200   0.70212100
H   0.42194400 -0.78231300   0.60243500
C -1.75783100   0.89014000   0.10188000
H -0.86923300   1.41098800   0.47159200
H -2.64379500   1.40936600   0.48537400
H -1.77069300   0.98412400 -0.99483100
Pd -1.28682600 -0.76617800   2.66274100

TS: Pd + C_6H_5–CH_3
E = -2019.61
H = -1937.92
G = -1964.87
$\boldsymbol{N_{imag}}$ = 1, -413.50 cm^{-1}

C	-1.16133700	1.44303900	-0.51131700
C	-2.45370400	0.86883400	-0.51002200
C	-2.64052900	-0.45822600	-0.10700700
C	-1.54626200	-1.24529800	0.27432800
C	-0.25815700	-0.69506700	0.24957300
C	-0.06100800	0.63159200	-0.14969900
H	-3.31040000	1.45980500	-0.82571800
H	-3.64498600	-0.87888900	-0.09735000
H	-1.69448300	-2.27993600	0.57680500
H	0.59963100	-1.30143600	0.53691500
H	0.94744000	1.03750600	-0.18426900
Pd	-1.06784000	3.38301800	0.07801100
C	-0.80360500	2.73658700	-2.00293800
H	-0.68417800	3.83737400	-2.07713800
H	-1.65127700	2.43574200	-2.61577000
H	0.11683800	2.26315800	-2.33961500

P: Pd + C_6H_5–CH_3
E = -2039.00
H = -1956.69
G = -1985.41
$\boldsymbol{N_{imag}}$ = 0

C	-1.23065200	1.37536900	-0.08560200
C	-2.48912100	0.78747300	-0.30209000
C	-2.64190600	-0.60015700	-0.16766800
C	-1.54565300	-1.40567700	0.16249400
C	-0.28910900	-0.82102100	0.35663000
C	-0.12309800	0.56581800	0.22368400
H	-3.34812100	1.39822600	-0.56805500
H	-3.62164900	-1.04818300	-0.32726500
H	-1.66818700	-2.48269000	0.25928900
H	0.57125900	-1.44106600	0.60437100
H	0.86294200	1.00252100	0.36263500
Pd	-1.02950000	3.34119900	-0.02763500
C	-0.89287900	3.61735400	-2.03870100
H	-0.74930100	4.70488800	-2.12180200
H	-1.82148200	3.30364500	-2.52210800
H	-0.03250500	3.07481900	-2.43753900

RC: Pd + H_2C=CH–CH_3
E = -1105.85
H = -1052.67
G = -1075.18
$\boldsymbol{N_{imag}}$ = 0

C	1.57185500	1.15112000	1.48866500
C	1.75211000	0.90284300	0.11172100

H	1.97670900	1.72561700	-0.56866700
C	1.73711200	2.51531500	2.12838800
H	1.69909900	0.30832600	2.17295000
H	2.04743700	-0.08733600	-0.23503500
Pd	-0.32898100	0.81421400	0.52119900
H	2.75378900	2.61508700	2.54248700
H	1.03852600	2.65985200	2.96114000
H	1.58519900	3.32546900	1.40682200

TS: Pd + $H_2C{=}CH{-}CH_3$

E = -1064.57

H = -1013.70

G = -1036.53

N_{imag} = 1, -468.31 cm^{-1}

C	0.15702800	-0.04681700	-0.06679700
C	2.14138800	-0.04586600	0.15628300
H	2.47511200	-0.59081600	-0.72530700
C	-0.35623700	-0.72516000	-1.11409400
Pd	0.99439200	1.75629300	-0.34519300
H	2.87884600	0.72936600	0.43904300
H	2.02577800	-0.71085500	1.01211700
H	-1.26724100	-1.31539600	-1.00107900
H	0.10836400	-0.73136300	-2.09746700
H	-0.29997400	-0.15169000	0.91979000

P: Pd + $H_2C{=}CH{-}CH_3$

E = -1082.92

H = -1031.61

G = -1056.21

N_{imag} = 0

C	0.23518500	0.17013700	0.20985600
C	3.16516700	-0.07946000	-0.34162200
H	3.24236400	0.80506000	0.29443100
C	-0.28945600	0.71236000	1.31377600
Pd	1.59676400	-1.24249300	0.23093700
H	4.02580700	-0.74845600	-0.19433300
H	3.04722000	0.19132600	-1.39417900
H	-1.15269700	1.38050200	1.24133500
H	0.08971400	0.52435900	2.31609600
H	-0.12923700	0.41704300	-0.79062500

RC: Pd + $HC{\equiv}C{-}CH_3$

E = -910.52

H = -872.14

G = -895.05

N_{imag} = 0

C	1.39522100	1.64340200	1.07907500
C	1.61356400	0.78971500	0.17533600
H	2.80726800	2.58019800	2.36080700
C	1.72711900	2.59506600	2.15944900
H	1.44095300	3.61824000	1.88749500

H	1.20069300	2.34043300	3.08733100
Pd	-0.42670000	1.06904600	0.18682400
H	2.24015900	0.15524800	-0.42734500

TS: Pd + HC≡C–CH$_3$

E = -872.16
H = -835.69
G = -858.36
***N*$_{imag}$** = 1, -430.29 cm^{-1}

C	0.21016500	-0.18445800	0.01930500
C	2.14375000	-0.00137700	0.09620200
H	2.37127500	-0.60700700	-0.77721200
C	-0.56004200	-1.13740500	0.08006800
Pd	0.73551800	1.66532500	-0.17247400
H	2.81368000	0.87938000	0.13339300
H	2.23218100	-0.55459100	1.02700300
H	-1.25537300	-1.94515700	0.14400400

P: Pd + HC≡C–CH$_3$

E = -888.06
H = -850.98
G = -874.71
***N*$_{imag}$** = 0

C	0.15485000	-0.01647400	0.01218100
C	3.02474200	-0.02761000	-0.12262200
H	3.09062600	0.52018900	0.81915600
C	-0.73487900	0.81834500	0.02934100
Pd	1.51272700	-1.35880200	-0.01309400
H	3.87293300	-0.71641200	-0.26601500
H	2.89152100	0.63801000	-0.97629600
H	-1.54122100	1.51822500	0.04692600

RC: PdCl$^-$ + H$_3$C–CH$_2$–CH$_3$

E = -1390.88
H = -1322.36
G = -1350.84
***N*$_{imag}$** = 0

C	1.51934300	0.02187500	-0.05128800
H	1.86406100	1.06402000	-0.05673100
C	-0.01825100	0.00826800	0.00001800
C	2.09469800	-0.71468000	-1.27870600
H	1.92617600	-0.43663800	0.86579900
H	-0.41574800	-1.01399900	0.04670500
H	-0.38996700	0.54833000	0.92201400
H	-0.44833000	0.48695100	-0.93056200
H	1.77182900	-1.76566900	-1.29634300
H	1.74816000	-0.24707100	-2.21012400
H	3.19458600	-0.70233000	-1.28141600
Pd	-1.48707500	1.88913300	-0.00837200
Cl	-2.92718300	3.75031000	-0.01649600

TS: PdCl$^-$ + $H_3C-CH_2-CH_3$

E = -1361.21

H = -1294.83

G = -1322.41

N_{imag} = 1, -484.94 cm^{-1}

C	-0.95860200	-1.99642100	0.00498600
C	0.94461300	-1.97107500	0.01210400
H	-1.00352500	-2.72967500	0.81743100
C	-1.45032000	-2.60957500	-1.31215200
H	-1.65587300	-1.17121300	0.31288000
H	1.69404900	-1.20902300	-0.31729600
H	1.20533800	-2.31433900	1.01792800
H	1.02562500	-2.80576700	-0.69217100
H	-2.46963000	-3.01615500	-1.19865700
H	-1.47794600	-1.85842700	-2.10905200
H	-0.80531800	-3.43573500	-1.65194000
Pd	-0.01596500	-0.00045800	-0.00880900
Cl	0.02605500	2.40136200	-0.05455300

P: PdCl$^-$ + $H_3C-CH_2-CH_3$

E = -1386.95

H = -1319.87

G = -1348.97

N_{imag} = 0

C	-1.14295800	1.73627600	0.33448200
C	1.35421800	1.58425300	-0.81105900
H	-0.69119200	2.70979100	0.57003100
C	-2.25717400	1.38804200	1.33102800
H	-1.52936200	1.77645600	-0.69416600
H	2.23375500	1.02027600	-1.16216300
H	0.78385000	1.93626400	-1.68327700
H	1.69540800	2.45661600	-0.23324400
H	-3.07503500	2.13491200	1.28593100
H	-2.69281400	0.40354100	1.12371000
H	-1.88486700	1.36573500	2.36250600
Pd	0.29669600	0.27246000	0.38378400
Cl	0.05107600	-1.84302000	1.60133700

RC: PdCl$^-$ + $C_6H_5-CH_3$

E = -2179.02

H = -2094.26

G = -2124.99

N_{imag} = 0

C	-2.13239200	-0.35092400	0.00860300
C	-3.19958400	-1.22403400	-0.16746300
C	-3.09177500	-2.59717100	0.18085700
C	-1.90814800	-3.09555300	0.70227000
C	-0.76724200	-2.24467800	0.86881500
C	-0.88350500	-0.85357800	0.52025600
H	-4.14280600	-0.84472600	-0.56382300
H	-3.95251900	-3.25343000	0.05111300

H	-1.82503100	-4.15014200	0.96542000
H	0.21698500	-2.70663200	0.96000300
H	0.01582700	-0.26136500	0.33739100
C	-2.24055700	1.11454800	-0.36457300
H	-2.07514100	1.75791400	0.50992400
H	-3.22968500	1.34978500	-0.77764300
H	-1.48424600	1.39319900	-1.11580000
Pd	-0.86522000	-1.05438900	2.67743800
Cl	-0.76423800	-0.40640500	4.97359400

TS: $PdCl^-$ + C_6H_5–CH_3

E = -2149.27

H = -2066.29

G = -2096.67

N_{imag} = 1, -432.29 cm^{-1}

C	-1.23975300	1.40267600	-0.56402300
C	-2.53069200	0.83317400	-0.37177600
C	-2.70623900	-0.54163300	-0.18883400
C	-1.61035400	-1.41809500	-0.22957700
C	-0.33212700	-0.88555700	-0.45841100
C	-0.15081800	0.48908900	-0.63994600
H	-3.39830300	1.49015100	-0.34460100
H	-3.70903000	-0.93374900	-0.01080000
H	-1.74976000	-2.48949400	-0.09026300
H	0.53419800	-1.54836100	-0.49284700
H	0.85064100	0.87585400	-0.82038700
Pd	-0.89280600	3.21278500	0.28167300
C	-1.16172000	2.81696700	-1.87928700
H	-1.05561200	3.91770300	-1.79684600
H	-2.11858400	2.59670700	-2.35529600
H	-0.34011900	2.42596400	-2.48175000
Cl	-0.46365200	4.47434500	2.25850400

P: $PdCl^-$ + C_6H_5–CH_3

E = -2165.50

H = -2081.77

G = -2113.98

N_{imag} = 0

C	-1.10179900	1.10249500	0.02619400
C	-2.44756800	0.84410800	-0.33177800
C	-2.88845200	-0.43665800	-0.69606000
C	-1.99158600	-1.51322700	-0.73805700
C	-0.64780500	-1.28429500	-0.40927500
C	-0.21469400	-0.00218100	-0.04005300
H	-3.17142800	1.65855700	-0.31604700
H	-3.93870200	-0.59461200	-0.94965500
H	-2.33118100	-2.50846900	-1.02488300
H	0.06874200	-2.10733500	-0.44388000
H	0.83749500	0.14116200	0.20745600
Pd	-0.43076100	2.83416500	0.79232500
C	-0.87275900	3.70867000	-1.01205600

H	-0.64127500	4.76725800	-0.85174400
H	-1.93057100	3.56932900	-1.26136800
H	-0.24189600	3.28233100	-1.79998300
Cl	0.48580900	4.50820900	2.28726400

RC: PdCl$^-$ + $H_2C{=}CH{-}CH_3$

E = -1231.97
H = -1177.46
G = -1203.45
N_{imag} = 0

C	1.42757600	-0.04090700	-0.08435300
Cl	0.74082000	4.32745900	-0.51605500
C	0.02205200	-0.02955000	0.05579700
C	2.14042200	-0.57395000	-1.31812400
H	2.42731900	-1.63604600	-1.19130600
H	1.50356900	-0.50228900	-2.20828800
Pd	0.70786400	1.95760300	-0.23849900
H	3.06101200	-0.01110900	-1.52420300
H	2.02953000	-0.09622800	0.82906100
H	-0.43446000	-0.09852600	1.04564800
H	-0.61610200	-0.36730500	-0.76535500

TS: PdCl$^-$ + $H_2C{=}CH{-}CH_3$

E = -1192.62
H = -1140.33
G = -1166.46
N_{imag} = 1, -454.60 cm^{-1}

C	-0.81669900	-1.88020900	-0.06651200
C	1.09438800	-2.04555200	0.02781600
Pd	0.09150400	-0.08920100	-0.13792500
C	-1.43814500	-2.49855700	-1.10635300
Cl	-0.20051100	2.27459600	-0.27941800
H	1.87606100	-1.34879400	0.39186300
H	1.00273700	-2.84171800	0.77178300
H	1.37508800	-2.46797500	-0.93892100
H	-2.36222500	-3.06166300	-0.95379700
H	-1.06814000	-2.43636800	-2.12882400
H	-1.22852000	-2.06631600	0.93339100

P: PdCl$^-$ + $H_2C{=}CH{-}CH_3$

E = -1193.29
H = -1141.70
G = -1168.86
N_{imag} = 0

C	-1.48550000	1.16720000	-0.02120000
C	1.29290000	1.73980000	-0.20560000
Pd	0.19640000	-0.01070000	-0.04590000
C	-1.73762200	1.76940900	1.16044200
Cl	-0.41860000	-2.38310000	0.13300000
H	2.24040000	1.46860000	-0.70430000
H	0.81520000	2.54280000	-0.78810000

```
H   1.52130000   2.12700000   0.80160000
H  -2.75732300   1.93097800   1.47746900
H  -0.91962000   2.08960800   1.78872900
H  -2.11918000   1.29500800  -0.88636700
```

RC: PdCl^{-}+ HC≡C–CH$_3$

E = -1036.82

H = -996.90

G = -1023.19

***N*$_{imag}$** = 0

```
C   1.32891700   0.16062600  -0.50454400
Cl  0.65064600   4.49597700  -0.32202300
C   0.15404700   0.11653400  -0.02930800
C   2.58716700  -0.44728300  -0.99867200
H   2.55011300  -1.54985500  -0.96662900
H   2.79271100  -0.14250900  -2.03475000
Pd  0.65515300   2.10989300  -0.27709700
H   3.44726600  -0.11731500  -0.39856800
H  -0.70116900  -0.43577400   0.32870200
```

TS: PdCl^{-}+ HC≡C–CH$_3$

E = -1005.74

H = -967.79

G = -993.88

***N*$_{imag}$** = 1, -417.07 cm^{-1}

```
C  -0.66813500  -1.84876400  -0.49675400
C   1.10089400  -1.99073600   0.00316400
Pd  0.01684800  -0.06190300  -0.18848400
C  -1.49974400  -2.72264100  -0.77800100
Cl -0.27286900   2.30239300  -0.11572900
H   1.83534900  -1.20341900   0.26506700
H   0.96840600  -2.65022200   0.86008500
H   1.46452900  -2.53705700  -0.86660400
H  -2.33123000  -3.33425300  -1.04784400
```

P: PdCl^{-}+ HC≡C–CH$_3$

E = -1024.57

H = -985.81

G = -1013.21

***N*$_{imag}$** = 0

```
C  -1.64578600   1.42719700   0.16646000
C   1.13018800   1.42427000  -0.19483100
Pd -0.32427500  -0.00354900  -0.03605000
C  -2.50620200   2.29667700   0.30076400
Cl  1.00109900  -1.99064100  -0.24451900
H   2.05546600   0.85312000  -0.30993700
H   0.91424100   2.04244300  -1.07061800
H   1.12746200   2.02895800   0.71609500
H  -3.28116800   3.02149400   0.41806400
```

RC: Pd(PH$_3$)$_2$ + H$_3$C–CH$_2$–CH$_3$

E = -2014.86
H = -1911.76
G = -1945.38
N_{imag} = 0

Pd	-2.34707800	0.10848200	0.51801400
C	-2.26506900	0.58196300	-4.86666100
C	-2.29682900	-0.69598800	-5.72488300
H	-2.38178900	0.34693400	-3.80051700
H	-1.31822800	1.12314300	-4.98776800
H	-3.07752200	1.26608900	-5.14656700
H	-1.45434600	-1.34438600	-5.44354200
C	-3.61265000	-1.48391300	-5.58861700
H	-2.13906000	-0.43079400	-6.78038800
H	-5.55854500	-0.33187600	0.20476000
H	0.70700800	-0.52376900	1.44648000
H	-5.17510000	0.64699900	2.03541000
H	-5.15072800	1.73899100	0.22874900
H	0.75487000	0.60140900	-0.33914600
H	0.34758600	-1.46856500	-0.40652100
P	-0.11476200	-0.32444300	0.30209500
P	-4.57790000	0.54447000	0.74797800
H	-3.60575900	-2.38806900	-6.21025600
H	-3.78161500	-1.79374300	-4.54874200
H	-4.47228400	-0.87343600	-5.89647900

TS: Pd(PH$_3$)$_2$ + H$_3$C–CH$_2$–CH$_3$

E = -1961.73
H = -1859.51
G = -1895.39
N_{imag} = 1, -416.72 cm^{-1}

Pd	1.05369600	1.96359400	-0.00727200
C	2.15516500	0.01601900	0.01844400
C	0.02003800	-0.05619400	0.05081500
H	2.91713400	0.67845000	-0.42725100
H	2.43335100	-0.27062400	1.03319000
H	2.05682800	-0.86113800	-0.61888100
H	-0.69617800	0.66237600	0.49007300
C	-0.49523400	-0.65988100	-1.25021300
H	0.20319600	-0.81862100	0.80829400
H	-1.04791000	2.92119500	-2.53243100
H	2.70781900	4.58009100	1.40507000
H	-0.61259200	4.57658900	-1.34594400
H	0.82861600	3.83812700	-2.66760700
H	1.34354500	3.73660500	2.74331800
H	3.22083300	2.85198900	2.45405400
P	2.13094600	3.29475500	1.63986000
P	-0.00505000	3.31736700	-1.63530800
H	-1.37210200	-1.29656100	-1.05048700
H	-0.79802700	0.10622200	-1.97103000
H	0.25472700	-1.29755900	-1.73559500

P: $Pd(PH_3)_2$ + $H_3C{-}CH_2{-}CH_3$

E = -1985.37

H = -1882.65

G = -1916.54

N_{imag} = 0

C	0.05430100	-0.10146100	0.05210300
C	2.91333000	0.00037900	0.14885400
Pd	1.44179500	1.54398800	0.02304900
H	0.52053200	-0.89411800	0.64501100
H	-0.84734700	0.22222400	0.59100700
C	-0.30923600	-0.60660000	-1.35093200
H	2.89595600	-0.37524600	1.17853200
H	3.92671100	0.34663300	-0.08867700
H	2.64544000	-0.80806400	-0.53786500
P	-0.40387600	3.00645200	-0.03575000
P	3.21098200	3.11318300	-0.02604700
H	-1.12398300	3.21925000	1.17318600
H	-1.53628800	2.66145800	-0.82388600
H	-0.36199600	4.37797700	-0.42156500
H	3.08980000	4.53469600	-0.02589100
H	4.19538300	3.04676700	0.99778400
H	4.12232600	3.04325900	-1.11552000
H	-1.02107100	-1.44763200	-1.29767700
H	-0.77969000	0.17138000	-1.96693000
H	0.57263300	-0.96412600	-1.89648600

RC: $Pd(PH_3)_2$ + $C_6H_5{-}CH_3$

E = -2787.35

H = -2668.29

G = -2704.44

N_{imag} = 0

C	-1.84339100	-2.65803800	-0.08728100
C	-0.52619500	-3.10968800	0.10758700
C	0.46520000	-2.88427500	-0.85435000
C	0.15689100	-2.20058200	-2.03624100
C	-1.15094700	-1.74775100	-2.24623000
C	-2.13847400	-1.97616200	-1.28100100
H	-0.27530500	-3.64656900	1.02193700
H	1.47732100	-3.24666500	-0.68212800
H	0.92537300	-2.02749400	-2.78711300
H	-1.40456800	-1.22023700	-3.16424600
H	-3.15419700	-1.62337000	-1.45760300
C	-2.90699500	-2.87434700	0.97072800
H	-2.93437700	-2.03344700	1.68055500
H	-2.71481000	-3.78519100	1.54977000
H	-3.90453300	-2.95638600	0.52333900
Pd	-3.09579800	0.50107800	3.77539900
P	-1.61117600	1.65444500	2.46988200
H	-0.32560900	1.12502800	2.17044100
H	-1.93243900	2.02109100	1.13366100

H	-1.15227000	2.94049200	2.86851300
P	-4.57540200	-0.58945100	5.12980300
H	-4.25641100	-1.84858200	5.71162200
H	-5.03564800	0.01144200	6.33450400
H	-5.86318200	-0.97319600	4.66097900

TS: $Pd(PH_3)_2$ + C_6H_5–CH_3

E = -2741.09
H = -2623.02
G = -2659.77
N_{imag} = 1, -400.27 cm^{-1}

C	-1.12049400	1.43162100	-0.40088000
C	-2.51146500	1.37568700	-0.15601400
C	-3.11672100	0.19388800	0.29317500
C	-2.36070900	-0.96857300	0.47801600
C	-0.98318200	-0.93457200	0.20841400
C	-0.37187200	0.24064600	-0.23320700
H	-3.12712200	2.25902400	-0.31445500
H	-4.18845900	0.18472400	0.48893900
H	-2.83385100	-1.88801600	0.81619400
H	-0.38037900	-1.83225100	0.34260200
H	0.69566200	0.23772000	-0.44711500
Pd	-0.22980000	3.32241300	-0.02153400
C	-0.72447800	2.47398500	-2.07870300
H	-1.59623200	3.06486200	-2.36794500
H	-0.77060600	1.50292500	-2.56530200
H	0.20934800	2.95511400	-2.39982700
P	0.34760700	3.02436700	2.27059200
H	0.92570100	1.78429400	2.67032000
H	1.21393100	3.83378600	3.06867800
H	-0.70346500	2.99045200	3.23270500
P	0.17633600	5.65203600	-0.43262700
H	1.36298100	6.33458400	-0.02981200
H	0.16735100	6.20866600	-1.74881400
H	-0.70889100	6.62647800	0.11499300

P: $Pd(PH_3)_2$ + C_6H_5–CH_3

E = -2759.42
H = -2640.23
G = -2677.47
N_{imag} = 0

C	-1.03746600	1.37465100	0.02925800
C	-2.40416400	1.09785800	0.22512400
C	-2.87026900	-0.22231500	0.32238200
C	-1.97810000	-1.29615200	0.22311000
C	-0.61697400	-1.03760000	0.02483900
C	-0.15325200	0.28353500	-0.07211800
H	-3.12405100	1.91228100	0.29630900
H	-3.93368100	-0.40826300	0.47145800
H	-2.33928200	-2.32057400	0.29490900
H	0.08888700	-1.86359400	-0.05958300

H	0.91022300	0.45280400	-0.23651400
Pd	-0.34402500	3.34298700	-0.07724800
C	-0.69878000	3.17090700	-2.17049600
H	-1.45589400	3.91764100	-2.44005800
H	-1.04717500	2.17610700	-2.44752100
H	0.23994200	3.39372800	-2.69258800
P	-0.06802700	3.18969700	2.28038300
H	-1.21471000	2.85168900	3.04617900
H	0.76792200	2.15106900	2.76826000
H	0.41401700	4.20576000	3.15922200
P	0.39899400	5.56411900	-0.41950900
H	1.74692400	5.93752700	-0.15329500
H	0.33122300	6.12550400	-1.72317400
H	-0.19204800	6.66550300	0.26241800

RC: $Pd(PH_3)_2$ + $H_2C{=}CH{-}CH_3$

E = -1830.77

H = -1741.81

G = -1776.48

N_{imag} = 0

Pd	-2.58967800	-0.11369400	0.40010600
C	-2.16663700	0.79781200	-4.85999900
C	-2.47702000	-0.12117400	-5.77951500
H	-2.60080200	-2.27739600	-5.81331000
H	-3.46063100	-1.51934700	-4.45139600
H	-4.13774900	-1.46480200	-6.09696300
P	-0.45645100	-0.82813000	-0.00117700
C	-3.20783300	-1.41117600	-5.51246000
P	-4.71936300	0.60145500	0.81121100
H	-5.83384100	-0.26517400	0.63288700
H	0.51271100	-0.85906900	1.04012800
H	-5.10133300	1.06138200	2.10243900
H	-5.27678300	1.69910200	0.09768700
H	0.37270100	-0.17081300	-0.95158900
H	-0.20035100	-2.14518400	-0.47476000
H	-2.43151900	0.67144900	-3.81015200
H	-1.63662200	1.71037900	-5.12498400
H	-2.18802500	0.05588900	-6.81880200

TS: $Pd(PH_3)_2$ + $H_2C{=}CH{-}CH_3$

E = -1787.82

H = -1699.94

G = -1734.57

N_{imag} = 1, -440.32 cm^{-1}

Pd	0.97524300	1.86118800	0.10296500
C	1.51682200	-0.18428800	0.88921200
C	0.20664700	0.06433600	-0.63344900
H	1.68660500	0.33026900	1.84670400
H	0.90260200	-1.05900600	1.10421400
H	2.45699000	-0.49202400	0.42969300
P	2.68325800	3.32886200	0.92525900

C	0.60852300	-0.42859300	-1.82307400
P	-0.63921700	3.32351600	-0.84403300
H	-1.93134600	2.80863900	-1.15583400
H	3.51760100	4.01807800	-0.00213000
H	-1.11788400	4.55917600	-0.30946700
H	-0.39387500	3.84137700	-2.14888800
H	2.42147500	4.48006200	1.72638800
H	3.76041000	2.86614900	1.74073000
H	-0.78749700	-0.19240000	-0.26047200
H	-0.07247000	-1.00995000	-2.44656800
H	1.61055800	-0.27073400	-2.21792800

P: Pd(PH_3)$_2$ + H_2C=CH–CH_3

E = -1803.49
H = -1714.64
G = -1749.59
N_{imag} = 0

C	0.18933400	-0.03142800	-0.03281400
C	2.98473400	0.00315200	0.13549300
Pd	1.52516200	1.55889100	-0.01928200
H	4.26815000	3.06236700	-0.97472400
H	4.18108700	3.08678400	1.14183600
C	-0.57707200	-0.45344000	-1.04638700
H	2.81334200	-0.51035500	1.08740100
H	4.01389300	0.38024700	0.10823800
H	2.83117300	-0.69651100	-0.69013600
P	-0.36829900	2.96114100	-0.18064000
P	3.27997200	3.13395300	0.04401600
H	-1.35622900	2.85432700	0.83301700
H	-1.23722100	2.74817900	-1.28272300
H	-0.36018100	4.38694700	-0.24442000
H	3.13254500	4.55111700	0.02043600
H	-1.23420600	-1.32205200	-0.94318100
H	-0.58298300	0.01986000	-2.02923100
H	0.14695600	-0.58152700	0.91321600

RC: Pd(PH_3)$_2$ + HC≡C–CH_3

E = -1635.49
H = -1561.30
G = -1595.19
N_{imag} = 0

Pd	-2.47819200	-0.38685500	0.29508500
C	-2.81364000	1.52078000	-5.38848500
C	-2.89642800	0.49580600	-4.75381700
H	-2.26416800	-1.48239500	-4.34118200
H	-2.78695000	-0.56231000	-2.91469800
H	-3.98952500	-1.18329600	-4.06494300
P	-0.30260700	-1.08244200	0.21596300
C	-2.98846400	-0.73976600	-3.98146300
P	-4.63520000	0.34196900	0.51500300
H	-5.73654300	-0.45223400	0.08862400

H	0.43002400	-1.28361600	1.41866200
H	-5.15983000	0.63826800	1.80325200
H	-5.07616300	1.54433200	-0.10380800
H	0.69656900	-0.30742200	-0.43614400
H	0.06555300	-2.32040000	-0.38233700
H	-2.74348800	2.42597900	-5.94807900

TS: Pd(PH$_3$)$_2$ + HC≡C–CH$_3$

E = -1596.20
H = -1522.56
G = -1557.04
N_{imag} = 1, -442.28 cm^{-1}

Pd	1.17605500	1.86029000	0.00130500
C	2.01167500	-0.27399100	0.34469700
C	0.45786900	0.13023200	-0.71274400
H	2.88901900	0.30691000	0.65410000
H	1.55565200	-0.76271800	1.20304400
H	2.33291000	-0.99076400	-0.40498100
P	2.25829700	3.38234700	1.51548300
C	-0.35998500	-0.60448300	-1.26231800
P	-0.21340600	3.25388600	-1.36179800
H	-1.60592300	2.99187900	-1.25581700
H	2.95588300	4.53640300	1.05491000
H	-0.34284600	4.67430900	-1.47708600
H	-0.12104800	3.00874700	-2.75862700
H	1.45746000	4.08642500	2.45915900
H	3.26163300	3.00002500	2.45665000
H	-1.08040900	-1.22218500	-1.75058900

P: Pd(PH$_3$)$_2$ + HC≡C–CH$_3$

E = -1616.53
H = -1541.64
G = -1575.55
N_{imag} = 0

C	0.23067200	0.16045700	-0.48681100
C	3.03426000	0.08554300	-0.12631100
Pd	1.57020600	1.63128600	-0.16485100
H	4.31722200	3.22925900	-0.77720400
H	4.13918600	2.93074100	1.32087700
C	-0.60585400	-0.70535800	-0.69419000
H	2.84813000	-0.48848400	0.78526200
H	4.05985100	0.47078800	-0.12811600
H	2.86708100	-0.54150600	-1.00338100
P	-0.34019600	3.05900200	-0.26639300
P	3.29261700	3.13885500	0.20101300
H	-1.38940200	2.77801400	0.64270000
H	-1.10322200	3.00370900	-1.45847200
H	-0.34855000	4.47794400	-0.11577800
H	3.09807500	4.53563300	0.38188100
H	-1.31746900	-1.48043800	-0.87692900

Table S9. Cartesian coordinates (in Å), energies (*E*, *H* and *G*, in kcal mol^{-1}), and number of imaginary vibrational frequencies (N_{imag}) of all stationary points and transition states of the C–Cl activation in the gas phase, computed at ZORA-BLYP/TZ2P.

R: H_3C–CH_2–Cl
E = -856.67
H = -813.01
G = -832.70
N_{imag} = 0

C	0.41078300	0.66979100	0.00000000
H	0.17003600	1.26182100	-0.88892100
H	1.49166100	0.47161200	0.00000000
H	0.17003600	1.26182100	0.88892100
C	-0.33850600	-0.65479800	0.00000000
Cl	-2.16258600	-0.40427400	0.00000000
H	-0.12851200	-1.24991100	-0.89128400
H	-0.12851200	-1.24991100	0.89128400

R: C_6H_5–Cl
E = -1626.75
H = -1567.33
G = -1589.82
N_{imag} = 0

C	0.00000000	0.00000000	-0.24265900
C	0.00000000	1.21899800	-0.92656000
C	0.00000000	1.21048600	-2.32647700
C	0.00000000	0.00000000	-3.02929100
C	0.00000000	-1.21048600	-2.32647700
C	0.00000000	-1.21899800	-0.92656000
H	0.00000000	2.15430000	-0.37383700
H	0.00000000	2.15625600	-2.86463700
H	0.00000000	0.00000000	-4.11695700
H	0.00000000	-2.15625600	-2.86463700
H	0.00000000	-2.15430000	-0.37383700
Cl	0.00000000	0.00000000	1.52968300

R: H_2C=CH–Cl
E = -670.05
H = -641.32
G = -660.16
N_{imag} = 0

C	-0.69463400	0.72835800	0.00000000
C	0.62924500	0.61376600	0.00000000
H	1.31730500	1.45366200	0.00000000
H	-1.14196600	1.71955800	0.00000000
H	-1.35629400	-0.13293400	0.00000000
Cl	1.47235400	-0.93247300	0.00000000

R: HC≡C-Cl

E = -464.11
H = -449.76
G = -467.02
N_{imag} = 0

C	0.00000000	0.00000000	0.97418800
C	0.00000000	0.00000000	-0.23293600
Cl	0.00000000	0.00000000	-1.88233800
H	0.00000000	0.00000000	2.04066200

RC: Pd + H_3C-CH_2-Cl

E = -870.17
H = -824.94
G = -849.96
N_{imag} = 0

C	0.22417400	-0.09970400	0.14261300
H	0.65457900	0.89152600	0.29308600
Cl	-1.60185400	0.12347700	0.56319700
C	0.40706600	-0.62645200	-1.26659900
H	0.57290200	-0.78952800	0.91467400
H	1.48268400	-0.71273300	-1.47768000
Pd	-2.81684300	1.39987200	-0.97253300
H	-0.03799200	0.05763400	-1.99795700
H	-0.04875900	-1.61461900	-1.38795000

TS: Pd + H_3C-CH_2-Cl

E = -857.57
H = -813.93
G = -838.34
N_{imag} = 1, -262.54 cm^{-1}

C	-0.01112200	-0.13841000	-0.01792500
Cl	2.09790700	-0.00977600	-0.07466100
H	-0.06176000	-0.69904400	0.91066400
C	-0.35420800	-0.89980600	-1.27194100
H	-0.54899300	0.82268300	0.06374100
H	0.22430100	-1.82590700	-1.35732100
Pd	1.11458700	2.10677400	0.29968900
H	-0.18939300	-0.29572900	-2.16940600
H	-1.42434400	-1.16090100	-1.23466000

P: Pd + H_3C-CH_2-Cl

E = -889.20
H = -845.05
G = -870.36
N_{imag} = 0

C	0.02839600	0.05916100	0.02496300
Cl	3.31204500	0.10316100	-0.26612400
H	0.15533300	0.64771600	-0.88764700
C	-0.11932500	0.89899500	1.27584500
H	-0.76652200	-0.70185000	-0.10469800
H	0.70835100	1.60742200	1.38024700

H	-0.18620500	0.29317600	2.18649500
Pd	1.54101000	-1.26756100	0.09010400
H	-1.05241400	1.48590700	1.20698300

RC: Pd + C_6H_5–Cl

E = -1638.26
H = -1577.14
G = -1605.53
N_{imag} = 0

C	-0.34940300	-1.29731500	-0.17572800
C	-1.58041600	-1.89759000	0.09152500
C	-1.59397800	-3.22198500	0.54593800
C	-0.39510700	-3.92263400	0.72513800
C	0.82696900	-3.29675200	0.44920800
C	0.85944800	-1.97343400	-0.00619400
H	-2.50477400	-1.34546800	-0.05445900
H	-2.54674000	-3.70296200	0.75794700
H	-0.41285800	-4.95167900	1.07681000
H	1.76194900	-3.83619800	0.58649800
H	1.80128900	-1.47949300	-0.22857400
Cl	-0.32215800	0.42018100	-0.72510400
Pd	-0.15439500	0.76673700	-3.03485300

TS: Pd + C_6H_5–Cl

E = -1634.71
H = -1574.38
G = -1601.10
N_{imag} = 1, -89.3 cm^{-1}

C	-0.95179500	0.07290100	0.77204100
C	-2.31292200	-0.25430800	0.71321500
C	-2.67186200	-1.56188200	0.36778800
C	-1.69151300	-2.51597400	0.06559500
C	-0.33793000	-2.15670400	0.10354700
C	0.04576400	-0.85543500	0.44612600
H	-3.06732900	0.49314900	0.93968200
H	-3.72600300	-1.82958900	0.33208300
H	-1.98030200	-3.52876600	-0.20534400
H	0.43000500	-2.88871500	-0.13851400
H	1.09237300	-0.56695600	0.46868600
Cl	-0.47846100	1.79943600	1.06513600
Pd	-0.46201600	0.88452600	3.30321700

P: Pd + C_6H_5–Cl

E = -1660.03
H = -1599.35
G = -1627.62
N_{imag} = 0

C	0.01964000	0.50949900	0.15321600
C	-1.16326900	-0.13430100	-0.23056800
C	-1.11256100	-1.50956200	-0.51283400
C	0.08394800	-2.21741100	-0.35624900

```
C    1.24006200  -1.56003300   0.08363000
C    1.21484500  -0.18571400   0.36316100
H   -2.10527700   0.40451400  -0.31002700
H   -2.01896400  -2.02104300  -0.83133500
H    0.11098400  -3.28651200  -0.55613900
H    2.16726900  -2.11338300   0.22098100
H    2.10433100   0.32264000   0.72427500
Pd  -0.03223500   2.45232500   0.09683200
Cl   0.33475600   3.06040300   2.24803300
```

RC: Pd + H_2C=CH-Cl

E = -701.85
H = -671.71
G = -694.26
N_{imag} = 0

```
C    1.60653700   1.10851800   1.48574800
C    1.72056100   0.93113300   0.09380100
H    1.93313600   1.77865100  -0.55470900
Cl   1.91599400   2.70116200   2.25703400
H    1.81291000   0.31092100   2.19579800
H    1.99490900  -0.05326600  -0.28604800
Pd  -0.32036200   0.87174300   0.68173400
```

TS: Pd + H_2C=CH-Cl

E = -686.12
H = -657.22
G = -680.00
N_{imag} = 1, -235.99 cm^{-1}

```
C    2.10504700   8.20445100   4.77595800
Cl   4.24992400   8.01079200   5.12394900
Pd   2.94134300  10.06209400   4.69106900
C    1.57768000   7.87268500   3.58763900
H    2.11315800   7.98511800   2.64922600
H    1.69643100   7.96545700   5.75452500
H    0.57661300   7.44102300   3.54615700
```

P: Pd + H_2C=CH-Cl

E = -703.33
H = -674.41
G = -697.25
N_{imag} = 0

```
C    0.12357200   0.08256700   0.12664700
Cl   3.32503900   0.16415800  -0.61821300
H    0.50554200   0.93225100   2.04550700
C   -0.17636700   0.79675200   1.20875000
Pd   1.60850500  -1.15127500   0.05066000
H   -1.15597000   1.27639400   1.28812600
H   -0.46099900   0.03179400  -0.79740900
```

RC: Pd + HC≡C–Cl

E = -499.35

H = -483.87

G = -506.31

N_{imag} = 0

```
C    1.42355500   1.59875700   1.22201000
C    1.59351800   0.94618100   0.14674000
Pd  -0.40821700   0.89529000   0.63788200
Cl   2.03758600   2.52812200   2.50365200
H    2.18266500   0.54135600  -0.65762400
```

TS: Pd + HC≡C–Cl

E = -482.50

H = -467.91

G = -490.43

N_{imag} = 1, -210.13 cm^{-1}

```
C    14.25412600   3.48224500   0.00000000
H    13.40452600   2.83627000   0.00000000
C    15.19791700   4.26073800   0.00000000
Cl   17.30216300   4.18264900   0.00000000
Pd   15.86294400   6.11678100   0.00000000
```

P: Pd + HC≡C–Cl

E = -499.20

H = -483.76

G = -507.13

N_{imag} = 0

```
C    0.11023200  -0.06099900   0.00000000
Cl   3.09592500   0.91163900   0.00000000
H   -1.06116900  -2.02312000   0.00000000
C   -0.46670100  -1.13543500   0.00000000
Pd   0.97885700   1.61033600   0.00000000
```

RC: $PdCl^-$ + $H_3C–CH_2–Cl$

E = -994.07

H = -947.52

G = -976.07

N_{imag} = 0

```
C    1.84608300  -0.14909500   0.20917300
H    2.36977900   0.75932600   0.51433900
Cl   0.04770700   0.10946800   0.88826700
C    1.80562600  -0.33722600  -1.29467800
H    2.21619800  -1.01812600   0.76136700
Cl  -2.90358300   3.04939500  -1.14637400
H    2.82563200  -0.33637700  -1.71188500
Pd  -1.30476600   1.58145700  -0.22668100
H    1.32046700  -1.28298900  -1.56456600
H    1.23247000   0.47929600  -1.75402700
```

TS: PdCl$^-$ + H_3C–CH_2–Cl

E = -982.76
H = -938.14
G = -965.18
N_{imag} = 1, -322.88 cm^{-1}

C	-1.04091900	-2.30446700	-0.05924400
Cl	1.20777200	-2.20619200	-0.02236900
H	-1.08151500	-2.97205600	0.79653300
C	-1.34915300	-2.89947100	-1.40296500
H	-1.44521500	-1.31604700	0.13741300
Cl	-0.00158000	2.32344100	0.26410800
H	-0.76839600	-3.81065300	-1.59287400
Pd	0.68652500	0.05073400	0.12822800
H	-2.42131200	-3.16713700	-1.45921800
H	-1.14426500	-2.18652200	-2.20768300

P: PdCl$^-$ + H_3C–CH_2–Cl

E = -1029.36
H = -983.18
G = -1011.34
N_{imag} = 0

C	-1.48308300	1.14807700	-0.03374200
Cl	1.54774100	2.17541500	-0.54858800
H	-1.38687200	1.95023800	-0.77291200
C	-1.80853400	1.67605800	1.35977800
H	-2.19285700	0.38483400	-0.36890400
Cl	-0.55905900	-1.93867500	0.37925800
Pd	0.32277200	0.20548900	-0.08267000
H	-1.06461800	2.40663500	1.69699000
H	-2.79392800	2.18113000	1.35111500
H	-1.85899600	0.86602000	2.09638300

RC: PdCl$^-$ + C_6H_5–Cl

E = -1766.67
H = -1704.12
G = -1735.49
N_{imag} = 0

C	-0.51515900	-1.33911200	-0.21314700
C	-0.76251500	-1.58338000	1.14279000
C	-0.50406900	-2.85609100	1.66476500
C	-0.00316900	-3.87459600	0.83791900
C	0.23737100	-3.60813400	-0.51819700
C	-0.01627400	-2.33984400	-1.05300200
H	-1.15106500	-0.78876500	1.77754600
H	-0.69449300	-3.05231200	2.71979500
H	0.19676200	-4.86345400	1.24720100
H	0.62615400	-4.39256100	-1.16705500
H	0.16070000	-2.10095300	-2.10159000
Cl	-0.84442100	0.30503300	-0.90080700
Pd	-0.44766300	0.75397900	-3.09608600
Cl	-0.13179800	1.49537700	-5.30315800

TS: PdCl⁻ + C_6H_5–Cl

E = -1768.92
H = -1707.79
G = -1738.97
N_{imag} = 1, -180.52 cm^{-1}

C	0.09279500	0.42557000	0.60530400
C	-1.15777000	-0.21579200	0.39505300
C	-1.17997500	-1.50441800	-0.15004400
C	0.01580700	-2.18648000	-0.43478000
C	1.24995700	-1.56898000	-0.17370900
C	1.30423200	-0.28252400	0.37769200
H	-2.07691700	0.30132400	0.65605900
H	-2.13940200	-1.98100000	-0.35309000
H	-0.01502000	-3.19295200	-0.84926700
H	2.17964600	-2.09432000	-0.39379600
H	2.25285100	0.17979500	0.63544100
Pd	0.20872800	2.07495800	-0.49306200
Cl	0.13194100	1.67598400	2.26682600
Cl	0.38016500	3.88591400	-2.11491100

P: PdCl⁻ + C_6H_5–Cl

E = -1801.89
H = -1740.11
G = -1769.75
N_{imag} = 0

C	0.11613100	0.45315600	-0.16521700
C	-1.11414500	-0.21827500	-0.24080700
C	-1.14683300	-1.61711400	-0.34142400
C	0.04187900	-2.35812000	-0.36672000
C	1.26789600	-1.68450200	-0.29020800
C	1.30919700	-0.28588000	-0.18953000
H	-2.04504800	0.34283300	-0.22140600
H	-2.10968800	-2.12617900	-0.39899300
H	0.01321500	-3.44427900	-0.44467200
H	2.20240200	-2.24663400	-0.30768300
H	2.26837600	0.22235600	-0.13064100
Pd	0.16745900	2.41290000	-0.01745700
Cl	0.10516900	2.52745200	2.33074300
Cl	0.24252200	2.85729300	-2.32368200

RC: PdCl⁻ + H_2C=CH–Cl

E = -835.07
H = -803.41
G = -829.49
N_{imag} = 0

C	1.49404000	0.16943300	-0.07627500
Cl	0.37748800	4.37349500	-0.47957500
C	0.10229300	-0.00777300	0.03964500
Cl	2.39542500	-0.52940700	-1.55036700
H	-0.33252500	-0.14583000	1.03287900

H -0.47264700 -0.43008000 -0.78429000
Pd 0.63199100 2.02111300 -0.25061600
H 2.18160700 0.09347100 0.76358400

TS: PdCl⁻ + H_2C=CH–Cl
E = -814.08
H = -783.81
G = -810.78
N_{imag} = 1, -314.22 cm^{-1}
C -0.82853500 -2.05969500 0.04689900
Cl 1.31773200 -2.40001100 0.31421500
Pd -0.24257300 -0.20861500 -0.18267100
C -1.35861800 -2.40223800 -1.16154500
Cl -0.02184200 2.22300400 -0.14794500
H -1.22042300 -2.36649900 1.01811000
H -2.38250000 -2.77439700 -1.23717700
H -0.78514500 -2.32465400 -2.08131700

P: PdCl⁻ + H_2C=CH–Cl
E = -845.08
H = -813.60
G = -841.00
N_{imag} = 0
C -1.46366200 0.89707200 0.25597300
Cl 1.65285500 1.99727900 0.06090300
Pd 0.27930900 0.09847600 -0.15120800
C -1.71495800 1.89441200 1.10924700
Cl -0.60161800 -2.06011200 -0.53067700
H -0.93871300 2.41844500 1.65992400
H -2.25546900 0.38414800 -0.29424900
H -2.74842500 2.22050500 1.26766900

RC: PdCl⁻ + HC≡C–Cl
E = -633.43
H = -616.18
G = -642.27
N_{imag} = 0
C 1.36010400 0.27557300 -0.42791700
Cl 0.55882500 4.52953900 -0.29678900
C 0.14574400 0.13997000 -0.07495300
Cl 2.83627000 -0.58657000 -0.82244800
Pd 0.66258600 2.15580000 -0.27507800
H -0.71601500 -0.45047900 0.18497000

TS: PdCl⁻ + HC≡C–Cl
E = -613.40
H = -597.48
G = -624.42
N_{imag} = 1, -262.17 cm^{-1}
C -0.84429900 -1.96506600 0.00000000
Cl -1.49933100 -0.09515700 0.00000000

Pd 1.01080300 -1.45575100 0.00000000
C -1.42681100 -3.06972900 0.00000000
Cl 3.38008200 -0.86270500 0.00000000
H -1.83250300 -4.05573400 0.00000000

P: $PdCl^-$ + HC≡C-Cl

E = -649.44
H = -632.25
G = -658.84
N_{imag} = 0

C	-1.38874800	1.11017700	0.10894500
Cl	1.66745300	1.94404700	-0.28803400
Pd	0.23007100	0.12274200	-0.05590900
C	-2.42445700	1.74567100	0.21711000
Cl	-0.70010200	-2.00677200	0.12452700
H	-3.33222800	2.29772600	0.30000000

RC: $Pd(PH_3)_2$ + H_3C-CH_2-Cl

E = -1613.24
H = -1532.87
G = -1566.19
N_{imag} = 0

Pd	-2.06913400	-0.00677600	-0.01281000
C	-2.01037700	0.40485400	-4.77614000
C	-2.74362900	-0.90777900	-5.00849600
H	-2.16914700	0.77343900	-3.75738500
H	-0.93258900	0.23980400	-4.91581400
H	-2.33481800	1.17315800	-5.48600700
H	-2.46708400	-1.67171700	-4.27884200
Cl	-4.56963900	-0.70945800	-4.83355900
H	-2.59603300	-1.29604700	-6.01872700
H	-4.90843600	-0.83433900	-1.39791900
H	0.71806100	-0.31543400	1.62445900
H	-5.19767900	-0.54927700	0.68124600
H	-5.06583100	1.11965800	-0.60922800
H	0.90595300	1.28183300	0.25806400
H	1.08862000	-0.70892700	-0.41446700
P	0.17911400	0.06261400	0.36280900
P	-4.33559900	-0.07160700	-0.34446300

TS: $Pd(PH_3)_2$ + H_3C-CH_2-Cl

E = -1585.70
H = -1505.85
G = -1540.86
N_{imag} = 1, -288.90 cm^{-1}

Pd	1.17124200	2.16645100	-0.16070300
Cl	2.32277700	-0.01352100	-0.47402600
C	0.04898900	-0.01957700	0.06190000
H	-1.54797500	-1.08537800	-0.82369000
H	0.04478200	-1.55379600	-1.46221400
H	-0.71088800	-0.02087900	-1.96608600

H	-0.48640300	0.87174200	0.42013800
C	-0.55590800	-0.70437500	-1.12637700
H	0.36353600	-0.65800400	0.87920600
H	-0.82224200	2.78195500	-2.83794400
H	2.62489600	4.57489000	1.68706600
H	-0.93911200	4.37306500	-1.49313100
H	0.77204800	4.13545400	-2.68156500
H	1.33174800	3.46973800	2.90342100
H	3.24561500	2.73033000	2.46131400
P	2.12402500	3.24125500	1.74017100
P	-0.00849100	3.33600200	-1.79862600

P: $Pd(PH_3)_2$ + $H_3C–CH_2–Cl$

E = -1620.74

H = -1538.55

G = -1572.67

N_{imag} = 0

C	-0.06616600	-0.01678700	0.06537600
Cl	3.10501100	-0.07023700	0.05169300
Pd	1.36356100	1.57672500	0.01862300
H	0.42014700	-0.70067300	0.76537000
H	-1.01772500	0.31794000	0.49732000
C	-0.25503800	-0.63468400	-1.31777600
H	0.69615600	-0.98372800	-1.73181400
H	-0.92894200	-1.50546200	-1.25180500
H	-0.70497400	0.06519700	-2.03396600
P	-0.41407900	2.95656100	0.01792000
P	3.23564100	3.11051900	-0.05001600
H	-1.23031200	3.01009600	1.17916700
H	-1.45564500	2.73564500	-0.92171700
H	-0.28305000	4.35834400	-0.17625100
H	3.16871700	4.53563700	-0.09808700
H	4.17256700	3.01367000	1.00818300
H	4.14085000	2.93994500	-1.12623300

RC: $Pd(PH_3)_2$ + $C_6H_5–Cl$

E = -2383.38

H = -2287.23

G = -2322.84

N_{imag} = 0

C	-0.81465700	-1.05151700	0.88717800
C	-0.79805500	-2.11753400	1.79172300
C	-0.48711000	-3.39966300	1.32279300
C	-0.19769600	-3.60912800	-0.03083400
C	-0.21954800	-2.52847500	-0.92036400
C	-0.52860600	-1.23982700	-0.46787100
H	-1.02394200	-1.94608900	2.84058900
H	-0.47244300	-4.23353000	2.02194800
H	0.04331900	-4.60758200	-0.38936200
H	0.00400300	-2.67759400	-1.97482900
H	-0.54476200	-0.40146300	-1.16039500

Cl	-1.20799200	0.57431000	1.47472300
Pd	-0.12539000	0.64251500	-4.32259100
P	-2.32796700	0.22737300	-4.77141700
H	-2.82513400	-1.10238400	-4.86741600
H	-3.35601300	0.70474300	-3.91172800
H	-2.91949000	0.69095600	-5.97948500
P	2.07709000	1.08960300	-3.90624000
H	3.02377900	0.04259900	-3.72792100
H	2.85329200	1.82971500	-4.84085400
H	2.47116500	1.83782400	-2.76238600

TS: $Pd(PH_3)_2$ + C_6H_5–Cl

E = -2363.46

H = -2267.33

G = -2304.80

N_{imag} = 1, -225.68 cm^{-1}

C	-0.12957900	0.54049000	0.47036700
C	-1.00597100	-0.43291100	-0.04610900
C	-0.47204500	-1.61179600	-0.56776900
C	0.91336300	-1.85031000	-0.54116600
C	1.76381300	-0.90162300	0.03408400
C	1.24918400	0.28563500	0.57764800
H	-2.07799600	-0.25699900	-0.05322700
H	-1.14525000	-2.35238400	-0.99698900
H	1.31604300	-2.77849100	-0.93984800
H	2.83504400	-1.08770300	0.09198200
H	1.90460900	0.99228700	1.07879700
Pd	-0.24423800	2.65455400	-0.13967600
Cl	-0.89911900	1.64765800	2.07071800
P	-0.92125100	2.36678900	-2.36903700
H	-2.01720100	1.52041200	-2.71105700
H	-1.31268000	3.44570600	-3.21362000
H	-0.02069600	1.78890600	-3.30871500
P	0.55877400	4.89878900	0.22827100
H	0.07514400	6.01442000	-0.51324400
H	0.45039400	5.53686500	1.49780100
H	1.93478600	5.21521100	0.03781500

P: $Pd(PH_3)_2$ + C_6H_5–Cl

E = -2392.03

H = -2293.48

G = -2330.82

N_{imag} = 0

C	-0.00495800	0.49437000	-0.24736000
C	-1.14454300	-0.31849700	-0.33301300
C	-1.01390600	-1.71553300	-0.36069100
C	0.25281200	-2.30779600	-0.30866400
C	1.39046600	-1.49746800	-0.22610200
C	1.26432200	-0.09993000	-0.19845700
H	-2.13974500	0.12138100	-0.35826600
H	-1.90698800	-2.33658700	-0.41631100

H	0.35213100	-3.39143600	-0.32723300
H	2.38110500	-1.94750900	-0.17647800
H	2.16124100	0.51151800	-0.11858100
Pd	-0.20169600	2.55622900	-0.08940300
Cl	-0.35082400	2.36799400	2.28416500
P	-0.03973700	2.49393000	-2.34775300
H	-0.99721600	1.73555400	-3.06610100
H	-0.09313400	3.67687500	-3.13496800
H	1.12782800	1.92923200	-2.91864800
P	-0.44292000	4.90749000	0.40191800
H	-0.47355700	5.97516400	-0.54444800
H	-1.60020700	5.27187200	1.13055200
H	0.52773200	5.45879700	1.27196000

RC: $Pd(PH_3)_2$ + $H_2C{=}CH{-}Cl$

E = -1426.42
H = -1360.97
G = -1394.27
N_{imag} = 0

Pd	-1.98200600	-0.03364200	0.28537000
C	-2.46133200	-0.26033800	-4.66136900
C	-2.98166200	-0.43287300	-5.87221300
P	0.26007500	0.35316900	0.49125600
H	1.21806700	-0.57625500	-0.00212700
P	-4.22921900	-0.41098300	0.10683700
H	-5.07287300	0.49136400	-0.59878200
Cl	-3.93702100	0.79275100	-6.70470100
H	0.86477200	1.50279900	-0.08948400
H	-4.73300400	-1.59020200	-0.50936000
H	0.85818000	0.50818900	1.77280500
H	-5.04628300	-0.49449000	1.26842800
H	-2.58817500	0.65645200	-4.09296500
H	-1.88404000	-1.06390800	-4.20975100
H	-2.87315100	-1.33602300	-6.46527100

TS: $Pd(PH_3)_2$ + $H_2C{=}CH{-}Cl$

E = -1410.86
H = -1345.01
G = -1379.90
N_{imag} = 1, -288.55 cm^{-1}

Pd	0.67196300	1.97077300	0.27536300
Cl	-0.14159200	0.22173300	1.85337200
C	-0.43274200	0.20853300	-0.23167400
H	-1.50125000	0.35225000	-0.36579200
H	1.34735300	-0.89406100	-0.68193800
H	-0.09970500	-1.12613000	-1.82924600
P	2.61878400	3.07206900	1.16636200
C	0.32070300	-0.65199600	-0.94182200
P	-0.35364000	3.38460300	-1.29405100
H	0.20065200	3.50751100	-2.60075500
H	2.65850800	4.47560200	1.40483300

H	-1.68588500	3.14670100	-1.74769500
H	-0.52493000	4.78560100	-1.09507100
H	3.13161600	2.70907600	2.44443600
H	3.87444700	3.02494600	0.49475500

P: $Pd(PH_3)_2$ + H_2C=CH-Cl

E = -1436.36
H = -1368.77
G = -1401.74
N_{imag} = 0

C	0.15482700	-0.00284700	-0.03793800
Cl	3.26651900	-0.01227200	0.24679800
Pd	1.49061700	1.56439200	-0.01102000
H	4.31795100	2.98894300	-0.89624400
H	4.11965100	3.11553300	1.23164400
C	-0.61890700	-0.40664500	-1.04656700
H	0.20680100	-0.56725600	0.89570200
H	-0.68162200	0.11018800	-2.00411700
H	-1.21220300	-1.32117400	-0.96431400
P	-0.35064900	2.85934700	-0.19718800
P	3.30319500	3.15802400	0.07602800
H	-1.33462300	2.77650500	0.81859900
H	-1.19828700	2.65384800	-1.31395700
H	-0.25955100	4.27679000	-0.26418800
H	3.18745700	4.57658500	-0.01943700

RC: $Pd(PH_3)_2$ + HC≡C-Cl

E = -1220.21
H = -1169.45
G = -1201.45
N_{imag} = 0

Pd	-2.36296700	-0.37281600	0.64779100
C	-2.82049500	2.75014700	-5.16509700
C	-2.34025200	1.66028600	-5.36265000
P	-0.10907300	-0.67312300	0.43340500
H	0.42557900	-1.86605000	-0.12926200
P	-4.61505300	-0.06104000	0.86308900
H	-5.26897500	1.02385100	0.21546600
Cl	-1.68331800	0.16996100	-5.62507600
H	0.67616500	0.21677500	-0.35094900
H	-5.54209000	-1.05694500	0.44581400
H	0.74840300	-0.65646900	1.56900200
H	-5.20561700	0.15708000	2.13930600
H	-3.24545100	3.71250000	-4.98979000

TS: $Pd(PH_3)_2$ + HC≡C–Cl

E = -1207.77
H = -1156.36
G = -1191.23
N$_{imag}$ = 1, -172.49 cm^{-1}

Pd	1.19466100	1.91284200	0.16483800
Cl	2.09233900	-0.50601300	0.32294700
C	0.37282200	0.00000000	-0.20565500
H	1.61206200	3.97681500	2.78455600
H	-1.71719700	-0.78149400	-0.69417500
H	3.44172800	2.97862200	2.46277700
P	2.31781700	3.39634900	1.69255500
C	-0.70409400	-0.56488800	-0.43414200
P	-0.05168000	2.96787900	-1.51558400
H	-0.34962400	2.26202600	-2.71430600
H	2.90858500	4.61641000	1.25786400
H	-1.40015400	3.29255800	-1.19958900
H	0.25341200	4.21502500	-2.13709200

P: $Pd(PH_3)_2$ + HC≡C–Cl

E = -1243.95
H = -1190.34
G = -1222.35
$\boldsymbol{N}_{imag}$ = 0

C	0.05489900	0.11851800	0.08206400
Cl	3.27328500	0.01903200	0.06308200
Pd	1.45373600	1.54545500	0.02889600
H	4.11443200	2.96714800	-1.13146200
H	4.14549000	3.04231200	1.02288200
C	-0.84763500	-0.70089400	0.11089000
H	-1.59132600	-1.46680000	0.13619400
H	-0.30286900	4.26319400	-0.04286500
H	3.04060700	4.51267000	-0.09152200
P	-0.40128200	2.84477700	-0.00079800
P	3.22102300	3.10024500	-0.04452000
H	-1.29354400	2.71664200	1.08646200
H	-1.30141200	2.65365100	-1.07237800

Table S10. Cartesian coordinates (in Å), energies (*E*, *H* and *G*, in kcal mol^{-1}), and number of imaginary vibrational frequencies (N_{imag}) of all stationary points and transition states of the C–H, C–Cl and C–C bond activation by Pd in the gas phase, computed at ZORA-BLYP-D3(BJ)/TZ2P.

R: $H_3C–CH_2–H$

E = -900.44
H = -852.16
G = -868.43
$\boldsymbol{N}_{imag}$ = 0

C	0.00000000	0.00000000	0.76969800
H	0.00000000	1.02232700	1.16809400
H	0.88536100	-0.51116400	1.16809400

```
H  -0.88536100  -0.51116400   1.16809400
C   0.00000000   0.00000000  -0.76969800
H  -0.88536100   0.51116400  -1.16809400
H   0.88536100   0.51116400  -1.16809400
H   0.00000000  -1.02232700  -1.16809400
```

R: $H_2C{=}CH{-}H$

E = -711.55
H = -677.90
G = -693.52
$\boldsymbol{N}_{\mathbf{imag}}$ = 0

```
C   0.00000000   0.00000000   0.66671100
C   0.00000000   0.00000000  -0.66671100
H   0.00000000   0.92571500  -1.23922400
H   0.00000000   0.92571500   1.23922400
H   0.00000000  -0.92571500   1.23922400
H   0.00000000  -0.92571500  -1.23922400
```

R: $HC{\equiv}C{-}H$

E = -510.47
H = -492.65
G = -507.61
$\boldsymbol{N}_{\mathbf{imag}}$ = 0

```
C   0.00000000   0.00000000  -0.60220200
C   0.00000000   0.00000000   0.60220200
H   0.00000000   0.00000000  -1.66958400
H   0.00000000   0.00000000   1.66958400
```

RC: Pd + $H_3C{-}CH_2{-}H$

E = -911.30
H = -862.98
G = -884.75
$\boldsymbol{N}_{\mathbf{imag}}$ = 0

```
C   0.21141800  -2.77562100   0.00000000
C   0.87834200  -1.38802600   0.00000000
H   0.50508700  -3.35077200  -0.88699400
H  -0.88045300  -2.69131200   0.00000000
H   0.50508700  -3.35077200   0.88699400
H   1.97160300  -1.44694700   0.00000000
H   0.60143600  -0.82809900  -0.93567800
H   0.60143600  -0.82809900   0.93567800
Pd -0.21920300   0.76691900   0.00000000
```

TS: Pd + $H_3C{-}CH_2{-}H$

E = -900.10
H = -853.96
G = -875.76
$\boldsymbol{N}_{\mathbf{imag}}$ = 1, -783.07 cm^{-1}

```
C   1.30496600  -0.64963400  -0.22874100
C   2.43449400   0.35104200   0.03892200
H   2.20224800   1.34601700  -0.35392000
```

H	2.64323200	0.45531000	1.10920000
H	3.35932000	0.00606000	-0.44984500
H	1.08209600	-0.75192000	-1.30306900
H	1.54453500	-1.64908900	0.14628300
H	0.31045200	-0.52922400	1.06239000
Pd	-0.69910600	0.05084000	0.01893100

P: Pd + $H_3C–CH_2–H$

E = -908.39
H = -861.63
G = -883.89
N_{imag} = 0

C	1.22590200	-0.62704200	-0.33034600
C	2.33420800	0.34561900	0.05722700
H	2.19636400	1.33661400	-0.38800900
H	2.40518500	0.46684900	1.14267400
H	3.30457100	-0.04221500	-0.29735300
H	1.14675500	-0.75997400	-1.42455300
H	1.33926400	-1.60617800	0.14659100
H	-0.42267800	-0.03917100	1.54412200
Pd	-0.68189200	-0.02657800	0.02364000

RC: Pd + $H_2C=CH–H$

E = -748.80
H = -713.62
G = -733.85
N_{imag} = 0

C	1.67933100	1.10374200	1.50540200
C	1.75116900	0.91997900	0.11178100
H	1.93110900	1.76703100	-0.54959400
H	1.80204200	2.09539100	1.93995600
H	1.85928600	0.26881200	2.18201400
H	1.98818800	-0.05897200	-0.30407800
Pd	-0.29532100	0.93501900	0.71296900

TS: Pd + $H_2C=CH–H$

E = -718.96
H = -687.48
G = -708.09
N_{imag} = 1, -697.03 cm^{-1}

C	1.27794500	-0.59099600	-0.13824500
C	2.31535600	0.24647200	0.01701800
H	0.18461100	-0.51081900	1.14094300
Pd	-0.65965500	-0.10322700	-0.12771500
H	2.21214400	1.25195700	0.41674000
H	3.32117900	-0.06444500	-0.27378200
H	1.43861700	-1.61984700	-0.46790800

P: Pd + $H_2C=CH–H$

E = -722.84
H = -690.64

G = -712.16
***N*_{imag}** = 0

C	1.28060400	-0.56334200	-0.12746400
C	2.32130300	0.26638800	-0.00670500
H	-0.57351300	-0.51042200	1.44191300
Pd	-0.59252500	-0.00134500	-0.01674600
H	2.23119800	1.30173600	0.31377000
H	3.33033200	-0.08412500	-0.24095400
H	1.39394000	-1.61940700	-0.38433800

RC: Pd + HC≡C-H
E = -547.20
H = -527.30
G = -547.31
N_{imag} = 0

C	1.94141900	1.09693100	1.38489900
C	2.00682900	0.93282100	0.13744300
Pd	0.00148500	1.04125600	0.65374300
H	2.33848500	1.21312600	2.37887100
H	2.50525700	0.81045800	-0.80901000

TS: Pd + HC≡C-H
E = -523.45
H = -506.51
G = -526.72
N_{imag} = 1, -575.93 cm^{-1}

C	0.36657300	0.34522200	0.00000000
C	1.39779800	1.00106200	0.00000000
H	0.13883000	-1.36726400	0.00000000
Pd	-1.25259500	-0.67231800	0.00000000
H	2.25665900	1.63596900	0.00000000

P: Pd + HC≡C-H
E = -524.66
H = -506.68
G = -527.23
N_{imag} = 0

C	0.37924800	0.34911700	0.00000000
C	1.39816100	1.01968200	0.00000000
H	-0.19189500	-1.80976600	0.00000000
Pd	-1.21863900	-0.68161600	0.00000000
H	2.25968900	1.65089500	0.00000000

R: $H_3C-CH_2-CH_3$
E = -1266.34
H = -1199.88
G = -1219.10
N_{imag} = 0

C	0.03545700	0.03003800	0.77542100
H	0.11394300	1.06848500	1.12329000
H	0.90417200	-0.51769700	1.16046600

H	-0.86322000	-0.40501700	1.23225100
C	-0.03621200	-0.03614000	-0.76055300
C	-1.24306300	0.72037500	-1.34364200
H	0.89054400	0.37448200	-1.18582800
H	-0.07820500	-1.08780000	-1.07783000
H	-1.21171300	1.78383400	-1.07246800
H	-2.18798300	0.30970900	-0.96415200
H	-1.26661000	0.65546300	-2.43829100

R: $H_2C{=}CH{-}CH_3$

E = -1080.72

H = -1029.04

G = -1047.97

$\boldsymbol{N_{imag}}$ = 0

C	-1.23082900	0.70440000	0.59750300
C	-1.16495000	0.47610500	-0.71713900
H	-1.19664900	1.32825200	-1.40046900
H	-1.31488000	1.71214200	0.99774800
H	-1.20309500	-0.11030100	1.32076500
C	-1.04745700	-0.87871900	-1.36308400
H	-0.13586500	-0.94605100	-1.97367700
H	-1.89166800	-1.06597700	-2.04188600
H	-1.02168500	-1.68107100	-0.61683000

R: $HC{\equiv}C{-}CH_3$

E = -883.28

H = -846.35

G = -864.05

$\boldsymbol{N_{imag}}$ = 0

C	0.00000000	0.00000000	1.01475400
C	0.00000000	0.00000000	-0.19291400
H	0.51199400	0.88680000	-2.04740300
H	-1.02398900	0.00000000	-2.04740300
H	0.51199400	-0.88680000	-2.04740300
C	0.00000000	0.00000000	-1.65258700
H	0.00000000	0.00000000	2.08117600

RC: Pd + $H_3C{-}CH_2{-}CH_3$

E = -1277.60

H = -1211.21

G = -1235.41

$\boldsymbol{N_{imag}}$ = 0

C	0.03246100	-0.05236700	-0.03517200
H	0.39776600	0.98233300	-0.01997800
C	-1.50877800	-0.04770700	0.01214700
C	0.59200300	-0.78208100	-1.27032100
H	0.41447400	-0.53080400	0.87827300
H	-1.92823200	-1.05980500	0.03858600
H	-1.86428500	0.46271000	0.94887200
H	-1.91506300	0.42998400	-0.92417500
H	0.25909100	-1.82808900	-1.29798600

H	0.25828300	-0.30066100	-2.19895900
H	1.68903200	-0.78073300	-1.26735600
Pd	-2.99775300	1.85442100	0.00577000

TS: Pd + $H_3C{-}CH_2{-}CH_3$

E = -1251.99
H = -1186.92
G = -1210.97
N_{imag} = 1, -475.67 cm^{-1}

C	0.04051200	-0.06590700	0.01655900
C	2.00643700	-0.01564100	0.10782400
H	-0.03229700	-0.69775400	0.90183900
C	-0.32745600	-0.81076600	-1.26886600
H	-0.70003900	0.76439300	0.18824100
H	2.78482800	0.77110700	-0.08162300
H	2.17436700	-0.43010000	1.10149500
H	2.13819600	-0.77147100	-0.66525800
H	-1.38107600	-1.12527900	-1.23546700
H	-0.19947900	-0.17943800	-2.15462800
H	0.28138300	-1.71354500	-1.40575200
Pd	1.00642200	1.89640100	-0.01416600

P: Pd + $H_3C{-}CH_2{-}CH_3$

E = -1279.32
H = -1213.58
G = -1239.02
N_{imag} = 0

C	0.01867100	-0.01079800	0.01654600
C	3.10118300	-0.05932100	-0.08146700
H	0.07961300	0.53101500	-0.93365800
C	-0.08676900	0.92438800	1.21721900
H	-0.82050600	-0.72871300	-0.03011400
H	3.96453400	-0.69749300	-0.31969100
H	2.93710000	0.67500000	-0.87486700
H	3.22500900	0.42453700	0.89240400
H	-1.00301400	1.53397600	1.13763400
H	-0.13645100	0.37918900	2.16600300
H	0.76012800	1.61803100	1.26649400
Pd	1.54260300	-1.35571100	0.03179500

RC: Pd + $H_2C{=}CH{-}CH_3$

E = -1116.68
H = -1063.48
G = -1086.00
N_{imag} = 0

C	1.57185500	1.15112000	1.48866500
C	1.75211000	0.90284300	0.11172100
H	1.97670900	1.72561700	-0.56866700
C	1.73711200	2.51531500	2.12838800
H	1.69909900	0.30832600	2.17295000
H	2.04743700	-0.08733600	-0.23503500

Pd -0.32898100 0.81421400 0.52119900
H 2.75378900 2.61508700 2.54248700
H 1.03852600 2.65985200 2.96114000
H 1.58519900 3.32546900 1.40682200

TS: Pd + $H_2C{=}CH{-}CH_3$
E = -1076.12
H = -1025.24
G = -1048.03
N_{imag} = 1, -464.83 cm^{-1}
C 0.15702800 -0.04681700 -0.06679700
C 2.14138800 -0.04586600 0.15628300
H 2.47511200 -0.59081600 -0.72530700
C -0.35623700 -0.72516000 -1.11409400
Pd 0.99439200 1.75629300 -0.34519300
H 2.87884600 0.72936600 0.43904300
H 2.02577800 -0.71085500 1.01211700
H -1.26724100 -1.31539600 -1.00107900
H 0.10836400 -0.73136300 -2.09746700
H -0.29997400 -0.15169000 0.91979000

P: Pd + $H_2C{=}CH{-}CH_3$
E = -1093.53
H = -1042.27
G = -1067.33
N_{imag} = 0
C 0.23518500 0.17013700 0.20985600
C 3.16516700 -0.07946000 -0.34162200
H 3.24236400 0.80506000 0.29443100
C -0.28945600 0.71236000 1.31377600
Pd 1.59676400 -1.24249300 0.23093700
H 4.02580700 -0.74845600 -0.19433300
H 3.04722000 0.19132600 -1.39417900
H -1.15269700 1.38050200 1.24133500
H 0.08971400 0.52435900 2.31609600
H -0.12923700 0.41704300 -0.79062500

RC: Pd + $HC{\equiv}C{-}CH_3$
E = -919.14
H = -880.76
G = -903.66
N_{imag} = 0
C 1.39522100 1.64340200 1.07907500
C 1.61356400 0.78971500 0.17533600
H 2.80726800 2.58019800 2.36080700
C 1.72711900 2.59506600 2.15944900
H 1.44095300 3.61824000 1.88749500
H 1.20069300 2.34043300 3.08733100
Pd -0.42670000 1.06904600 0.18682400
H 2.24015900 0.15524800 -0.42734500

TS: Pd + HC≡C–CH$_3$

E = -881.34
H = -844.87
G = -867.48
N_{imag} = 1, -437.01 cm^{-1}

C	0.21016500	-0.18445800	0.01930500
C	2.14375000	-0.00137700	0.09620200
H	2.37127500	-0.60700700	-0.77721200
C	-0.56004200	-1.13740500	0.08006800
Pd	0.73551800	1.66532500	-0.17247400
H	2.81368000	0.87938000	0.13339300
H	2.23218100	-0.55459100	1.02700300
H	-1.25537300	-1.94515700	0.14400400

P: Pd + HC≡C–CH$_3$

E = -896.57
H = -859.50
G = -883.46
N_{imag} = 0

C	0.15485000	-0.01647400	0.01218100
C	3.02474200	-0.02761000	-0.12262200
H	3.09062600	0.52018900	0.81915600
C	-0.73487900	0.81834500	0.02934100
Pd	1.51272700	-1.35880200	-0.01309400
H	3.87293300	-0.71641200	-0.26601500
H	2.89152100	0.63801000	-0.97629600
H	-1.54122100	1.51822500	0.04692600

R: H$_3$C–CH$_2$–Cl

E = -863.03
H = -819.36
G = -839.05
N_{imag} = 0

C	0.41078300	0.66979100	0.00000000
H	0.17003600	1.26182100	-0.88892100
H	1.49166100	0.47161200	0.00000000
H	0.17003600	1.26182100	0.88892100
C	-0.33850600	-0.65479800	0.00000000
Cl	-2.16258600	-0.40427400	0.00000000
H	-0.12851200	-1.24991100	-0.89128400
H	-0.12851200	-1.24991100	0.89128400

R: H$_2$C=CH–Cl

E = -674.98
H = -646.23
G = -665.08
N_{imag} = 0

C	-0.69463400	0.72835800	0.00000000
C	0.62924500	0.61376600	0.00000000
H	1.31730500	1.45366200	0.00000000
H	-1.14196600	1.71955800	0.00000000

```
H  -1.35629400  -0.13293400   0.00000000
Cl  1.47235400  -0.93247300   0.00000000
```

R: HC≡C–Cl

E = -467.27
H = -452.90
G = -470.17
N_{imag} = 0

```
C   0.00000000   0.00000000   0.97418800
C   0.00000000   0.00000000  -0.23293600
Cl  0.00000000   0.00000000  -1.88233800
H   0.00000000   0.00000000   2.04066200
```

RC: Pd + $H_3C–CH_2–Cl$

E = -881.21
H = -835.97
G = -860.98
N_{imag} = 0

```
C   0.22417400  -0.09970400   0.14261300
H   0.65457900   0.89152600   0.29308600
Cl -1.60185400   0.12347700   0.56319700
C   0.40706600  -0.62645200  -1.26659900
H   0.57290200  -0.78952800   0.91467400
H   1.48268400  -0.71273300  -1.47768000
Pd -2.81684300   1.39987200  -0.97253300
H  -0.03799200   0.05763400  -1.99795700
H  -0.04875900  -1.61461900  -1.38795000
```

TS: Pd + $H_3C–CH_2–Cl$

E = -868.78
H = -825.12
G = -849.43
N_{imag} = 1, -261.72 cm^{-1}

```
C  -0.01112200  -0.13841000  -0.01792500
Cl  2.09790700  -0.00977600  -0.07466100
H  -0.06176000  -0.69904400   0.91066400
C  -0.35420800  -0.89980600  -1.27194100
H  -0.54899300   0.82268300   0.06374100
H   0.22430100  -1.82590700  -1.35732100
Pd  1.11458700   2.10677400   0.29968900
H  -0.18939300  -0.29572900  -2.16940600
H  -1.42434400  -1.16090100  -1.23466000
```

P: Pd + $H_3C–CH_2–Cl$

E = -899.93
H = -855.77
G = -880.82
N_{imag} = 0

```
C   0.02839600   0.05916100   0.02496300
Cl  3.31204500   0.10316100  -0.26612400
H   0.15533300   0.64771600  -0.88764700
```

C -0.11932500   0.89899500   1.27584500
H -0.76652200 -0.70185000 -0.10469800
H   0.70835100   1.60742200   1.38024700

RC: Pd + H_2C=CH–Cl

E = -711.29
H = -681.13
G = -703.68
N_{imag} = 0

C   1.60653700   1.10851800   1.48574800
C   1.72056100   0.93113300   0.09380100
H   1.93313600   1.77865100 -0.55470900
Cl 1.91599400   2.70116200   2.25703400
H   1.81291000   0.31092100   2.19579800
H   1.99490900 -0.05326600 -0.28604800
Pd -0.32036200   0.87174300   0.68173400

TS: Pd + H_2C=CH–Cl

E = -695.77
H = -666.89
G = -689.76
N_{imag} = 1, -248.76 cm^{-1}

C   2.10504700   8.20445100   4.77595800
Cl 4.24992400   8.01079200   5.12394900
Pd 2.94134300 10.06209400   4.69106900
C   1.57768000   7.87268500   3.58763900
H   2.11315800   7.98511800   2.64922600
H   1.69643100   7.96545700   5.75452500
H   0.57661300   7.44102300   3.54615700

P: Pd + H_2C=CH–Cl

E = -712.27
H = -683.35
G = -706.16
N_{imag} = 0

C   0.12357200   0.08256700   0.12664700
Cl 3.32503900   0.16415800 -0.61821300
H   0.50554200   0.93225100   2.04550700
C -0.17636700   0.79675200   1.20875000
Pd 1.60850500 -1.15127500   0.05066000
H -1.15597000   1.27639400   1.28812600
H -0.46099900   0.03179400 -0.79740900

RC: Pd + HC≡C–Cl

E = -506.70
H = -491.17
G = -513.58
N_{imag} = 0

C   1.42355500   1.59875700   1.22201000
C   1.59351800   0.94618100   0.14674000
Pd -0.40821700   0.89529000   0.63788200

Cl	2.03758600	2.52812200	2.50365200
H	2.18266500	0.54135600	-0.65762400

TS: Pd + HC≡C-Cl

E = -489.82

H = -475.24

G = -497.80

N_{imag} = 1, -217.10 cm^{-1}

C	14.25412600	3.48224500	0.00000000
H	13.40452600	2.83627000	0.00000000
C	15.19791700	4.26073800	0.00000000
Cl	17.30216300	4.18264900	0.00000000
Pd	15.86294400	6.11678100	0.00000000

P: Pd + HC≡C-Cl

E = -505.94

H = -490.48

G = -513.82

N_{imag} = 0

C	0.11023200	-0.06099900	0.00000000
Cl	3.09592500	0.91163900	0.00000000
H	-1.06116900	-2.02312000	0.00000000
C	-0.46670100	-1.13543500	0.00000000
Pd	0.97885700	1.61033600	0.00000000
